# Identification of a critical role for ZIKV capsid α3 in virus assembly and its genetic interaction with M protein

**DOI:** 10.1371/journal.pntd.0011873

**Published:** 2024-01-02

**Authors:** Anastazia Jablunovsky, Anoop Narayanan, Joyce Jose

**Affiliations:** 1 Department of Biochemistry and Molecular Biology, The Pennsylvania State University, University Park, Pennsylvania, United States of America; 2 The Huck Institutes of the Life Sciences, The Pennsylvania State University, University Park, Pennsylvania, United States of America; Solena Ag, UNITED STATES MINOR OUTLYING ISLANDS

## Abstract

Flaviviruses such as Zika and dengue viruses are persistent health concerns in endemic regions worldwide. Efforts to combat the spread of flaviviruses have been challenging, as no antivirals or optimal vaccines are available. Prevention and treatment of flavivirus-induced diseases require a comprehensive understanding of their life cycle. However, several aspects of flavivirus biogenesis, including genome packaging and virion assembly, are not well characterized. In this study, we focused on flavivirus capsid protein (C) using Zika virus (ZIKV) as a model to investigate the role of the externally oriented α3 helix (C α3) without a known or predicted function. Alanine scanning mutagenesis of surface-exposed amino acids on C α3 revealed a critical C_N67_ residue essential for ZIKV virion production. The C_N67A_ mutation did not affect dimerization or RNA binding of purified C protein *in vitro*. The virus assembly is severely affected in cells transfected with an infectious cDNA clone of ZIKV with C_N67A_ mutation, resulting in a highly attenuated phenotype. We isolated a revertant virus with a partially restored phenotype by continuous passage of the C_N67A_ mutant virus in Vero E6 cells. Sequence analysis of the revertant revealed a second site mutation in the viral membrane (M) protein M_F37L_, indicating a genetic interaction between the C and M proteins of ZIKV. Introducing the M_F37L_ mutation on the mutant ZIKV C_N67A_ generated a double-mutant virus phenotypically consistent with the isolated genetic revertant. Similar results were obtained with analogous mutations on C and M proteins of dengue virus, suggesting the critical nature of C α3 and possible C and M residues contributing to virus assembly in other *Aedes*-transmitted flaviviruses. This study provides the first experimental evidence of a genetic interaction between the C protein and the viral envelope protein M, providing a mechanistic understanding of the molecular interactions involved in the assembly and budding of *Aedes*-transmitted flaviviruses.

## Author summary

Zika virus and other disease-causing flaviviruses constitute a significant threat to global human health. The development of effective antiviral strategies targeting flaviviruses has been hindered by a lack of evidence regarding their life cycle, particularly virion assembly. Here, we identified a critical Asn 67 residue in the α3 helix of the C protein mediating ZIKV assembly. We also isolated a compensatory mutation in the M protein, M_F37L,_ that rescues the assembly defect caused by the C_N67A_ mutation, revealing the first evidence of a genetic interaction between C and M protein. Using further molecular genetic analyses, we show that analogous resides in C and M proteins are also required for dengue virus (DENV) assembly, indicating a functional role of this interaction that is conserved among flaviviruses. The data reported here contribute to an increasing collection of evidence pointing toward an interaction between capsid and the external structural proteins of flaviviruses. Our findings describing the genetic interaction between C and M observed in cryo-EM structures but experimentally unproven significantly advance our understanding of *Aedes*-transmitted flavivirus assembly, which will be instrumental in designing assembly-specific flavivirus therapeutics and antivirals.

## Introduction

The Flavivirus genus in the *Flaviviridae* family is comprised of more than 70 viruses, including mosquito-borne viruses such as Japanese encephalitis virus (JEV), ZIKV, and dengue virus (DENV), which infects an estimated 100 million people annually, as well as tick-borne viruses like Powassan virus (POWV) and tick-borne encephalitis virus (TBEV). Many flaviviruses cause severe diseases, including hemorrhagic fever, congenital abnormalities, acute flaccid paralysis, and fatal encephalitis in humans [1,2]. A 2022 outbreak of JEV in Australia, infecting swine and causing human fatalities, was declared a communicable disease incident of national significance and is now considered a major global concern [3]. ZIKV gained public interest in 2014 when an epidemic in South America was linked to neurological dysfunction, intrauterine growth retardation, and severe birth defects [4,5]. Vaccination against flaviviruses is also complicated by high mutation rates and immunological factors, including antibody-dependent enhancement; even the currently approved vaccines against West Nile Virus, JEV, and DENV are suboptimal [6–8]. The extensive global spread and epidemic transmission of emerging and reemerging flaviviruses highlights a need for countermeasures targeting structural and functional aspects of virus infection [2].

The functional mechanisms of flavivirus assembly have been determined from the cryo-EM structures of virions and atomic structures of the component proteins [9]. Mature flaviviruses are comprised of an icosahedrally arranged outer shell containing envelope (E) and membrane (M) transmembrane proteins arranged on a host-derived lipid bilayer surrounding an inner core of genomic RNA and C protein [10]. The ~11 kb positive-sense RNA genome encodes a single polyprotein that is processed co-translationally by viral and host proteases into three structural [C, pre-Membrane (prM), and E] and seven non-structural proteins [NS1, NS2A, NS2B, NS3, NS4A, NS4B, and NS5] [11]. The non-structural proteins modify the host endoplasmic reticulum (ER) to form replication factories and vesicle packets similar to other positive-strand RNA viruses and replicate the genomic RNA with a double-stranded RNA intermediate [12–15]. Virus assembly initiates when the positive-strand genomic RNA bound to C protein forms a nucleocapsid, followed by its envelopment by prM and E proteins arranged as trimeric spikes of prM/E heterodimers on the ER membrane and budding into the ER lumen as an immature virus [12]. The 180 prM/E protomers form 60 trimeric spikes that arrange icosahedrally in the immature virus surface lattice, as observed in cryo-EM reconstructions [16,17]. The pr domain capping the fusion loop of E protein is cleaved by host furin during maturation, which is a pre-requisite for viral infectivity and occurs in the trans-Golgi compartment. The low pH environment of late Golgi induces rearrangement of the trimeric spike into a smooth immature virus structure, allowing the exposure of the furin cleavage site [16–19]. The cleavage of the pr domain by host furin at low pH leads to the stabilization of the smooth mature particle with 90 head-to-tail dimers of M/E in an icosahedral herringbone-like arrangement with cleaved pr still attached to E [20–22]. The mature virus is released into the extracellular milieu via the secretory pathway, and the neutral pH causes the shedding of pr, allowing the virion to mediate acidic pH-triggered membrane fusion upon entry into a new cell [22].

The nucleocapsid organization has not been detected in any mature flavivirus cryo-EM structures, which have resolved the icosahedrally arranged M and E proteins to atomic resolution [10,17,23–25]. However, a partially ordered nucleocapsid shell, including a density connecting the outer envelope to the internal core, has been observed in a 9-Å cryo-EM structure of immature ZIKV and in ~9.5-Å resolution antibody-stabilized structures of DENV and ZIKV [26,27]. Additionally, the presence of a nucleocapsid core was identified from an immature Kunjin virus (KUNV) cryo-EM structure at ~20-Å resolution obtained using asymmetric reconstruction [28]. These immature virus structures were generated using viruses purified from ammonium chloride-treated cells that did not encounter low pH in the late Golgi [29]. It is interesting to note that core density is not observed in the 7.8-Å resolution cryo-EM structure of immature Spondweni virus (SPOV) and 4.4-Å cryo-EM structure of Binjari virus (BinJV), which were both determined using immature virus particles selected from mature virus preparations [16,17]. Therefore, it is conceivable that there are transient interactions between C and prM/E proteins in the immature virus, which are possibly lost during virus maturation, leading to a rearrangement of the nucleocapsid core in the mature virions.

Despite currently available high-resolution structures of mature and immature flaviviruses, the mechanism of nucleocapsid formation and interactions of C protein with the viral envelope leading to virus assembly and budding are not well understood. Although hydrophobic interactions between the membrane-anchored prM/E proteins and the nucleocapsid that mediate virus assembly have been proposed, finding evidence for this link has been challenging due to the likely transient nature of potential interactions [2,26,27]. Transmembrane domains of E protein have been shown to contribute to the formation of nucleocapsid integration into the budding viral envelope in TBEV assembly [30]. Similarly, using the cryo-EM structure of ZIKV and structure-based mutagenesis, we have previously identified a conserved W474 residue on the E transmembrane domain interacting with the lipid pocket factor required for ZIKV assembly [31]. Interactions between prM and E are sufficient for budding as virus-like particles can be produced from the expression of prM and E, and subviral particles containing only prM and E are regularly found during virus infection [32–34]. Therefore, the mechanism orchestrating the nucleocapsid incorporation into budding virus particles remains undetermined, exacerbated by the lack of data regarding the C protein region involved in the formation of the nucleocapsid core that packages the viral genomic RNA. Furthermore, a packaging signal has not been detected in flavivirus genomic RNA, and the proposed interactions between C protein and genomic RNA occur in a nonspecific manner through electrostatic interactions [35]. Flavivirus assembly is also tightly coupled to RNA replication, with electron tomography studies showing nucleocapsid formation and virus budding into the ER lumen occur in close proximity to the RNA exit sites of replication vesicles in the ER [12–14]. Evidence supporting a replication-coupled assembly mechanism in flaviviruses has been obtained from non-structural protein NS2A interacting with the 3′ untranslated region (UTR) and has been proposed as an assembly chaperone that may provide the specificity for genome RNA packaging [9,36–38]. Although mutagenesis studies have characterized critical C protein residues in RNA binding, dimerization, and lipid droplet binding, thus far, a specific budding defective C protein mutation has not been identified to implicate a C protein region involved in virion formation [39–41].

The X-ray crystal and NMR structures of flavivirus C proteins expressed and purified from bacteria have shown the structure as a homodimer, with each monomer consisting of four α-helices (α1–α4) and an intrinsically disordered N-terminal region that remains unresolved [22,42–46]. The ZIKV C protein is a small 104 amino acid protein, and the structure of residues 23−98 has been determined by both X-ray crystallography and NMR [43,45]. The first alpha helix α1 (residues 37–40) forms the top level of the protein, alpha helices α2 (residues 44–56) and α3 (residues 63–71) and their connecting loop forms the middle layer, and alpha helix α4 (residues 74–97) which is oriented perpendicular to the α3 helix forms the bottom layer. The C protein dimerization is mediated by interactions of antiparallel α2 and α4 helices of the two subunits. There is a hydrophobic cleft between α1 and α2 helices in the C dimer, which has been proposed to interact with the membranes; however, this cleft is obstructed by the unstructured N-terminal domain in ZIKV [42,43]. The α4 helix contains several positively charged residues that are proposed to interact with RNA [47,48].

While several studies have characterized the structural importance of α1, α2, and α4 helices of flavivirus C protein [39,40,49,50], as well as the transmembrane anchor helix α5 [27], few have focused on the importance of the α3 helix, which forms the outer edge of the C protein and contains multiple surface-exposed residues in the dimeric structure. In this study, we determined that the α3 helix of ZIKV C protein is critical for virus assembly and budding, replacing a surface-exposed residue C_N67_ from the α3 with alanine inhibited virus production without affecting dimerization or RNA binding *in vitro*. We identified a second site compensatory mutation in the M protein, M_F37L_, which rescues the lethal phenotype of the C_N67A_ mutant. Further mutational analyses of DENV and characterization of DENV residues C_K67_ and M_L37_ in structurally similar positions in C α3 and M proteins revealed a significant reduction in virus titer and plaque sizes, confirming the importance of these residues across *Aedes*-transmitted flaviviruses. This study demonstrates the critical role of α3 in virus assembly and provides the first evidence for a genetic interaction of C protein with a membrane-anchored structural protein in mediating virus budding, a crucial step in flavivirus morphogenesis.

## Results

### Alanine scanning of C α3 identifies asparagine 67 as a critical residue for ZIKV production

**Fig 1:**
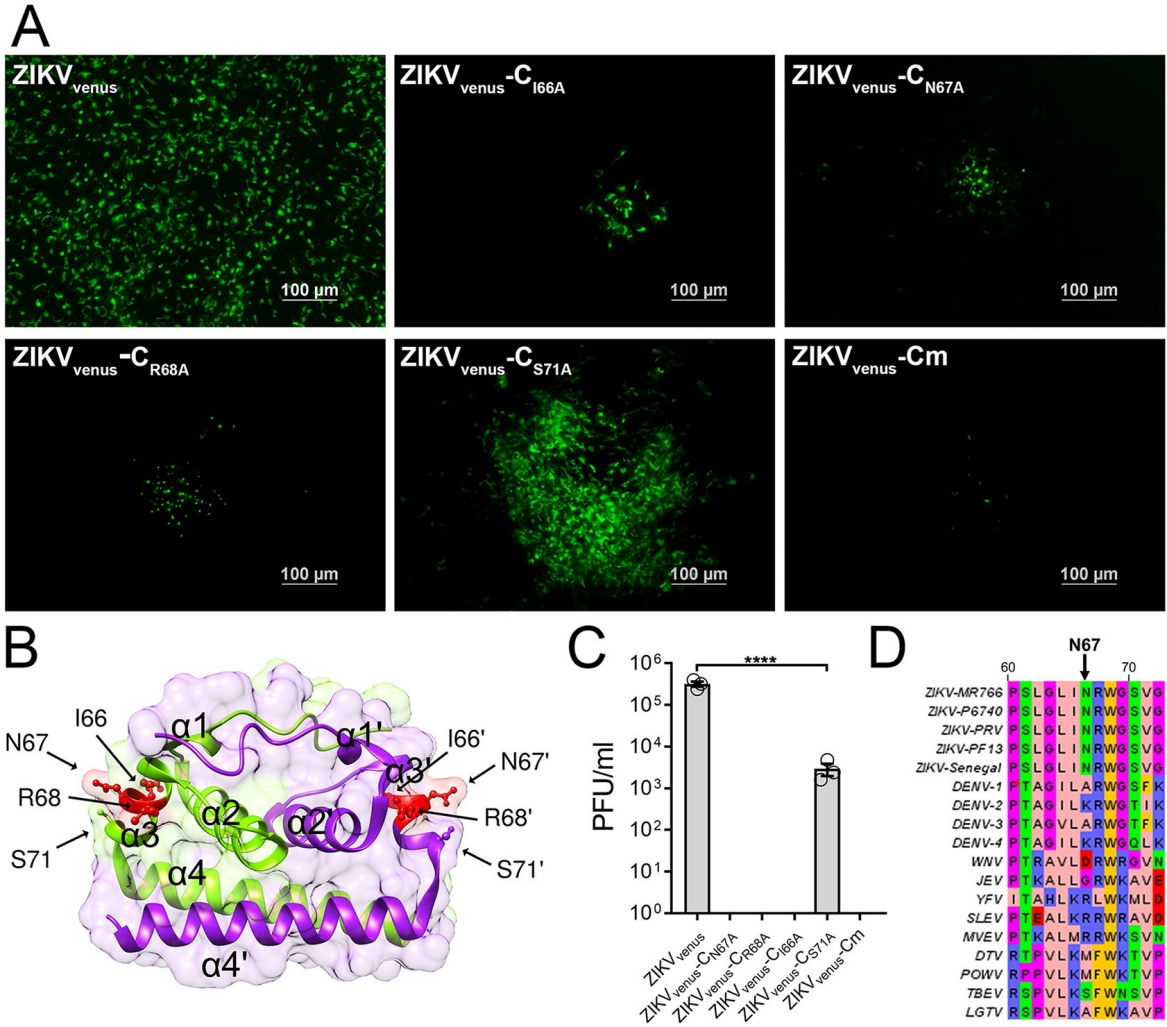
Mutational analysis of ZIKVC α3. (A) Micrographs showing fluorescent clusters of Vero E6 cells infected with wild-type ZIKV_venus_ or ZIKV_venus_ containing mutant C protein I66A(ZIKV_venus_-C_I66A_), N67A(ZIKV_venus_-C_N67A_), R68A(ZIKV_venus_-C_R68A_), S71A(ZIKV_venus_-C_S71A_), and combination mutant INRWSG/AAAWAG (ZIKV_venus_-Cm), showing virus spread. Images were acquired at 5 d.p.i. (B) The crystal structure of ZIKV C dimer (PDB:5YGH) was generated using UCSF Chimera software. Chain A is green, and chain B is purple with α1–4 labeled on the structure. Residues selected for mutational analysis are depicted as ball and stick. (C) Virus titers of wild-type and mutant ZIKV_venus_ (passage 0) as determined by plaque assay in Vero E6 cells. Column labels correspond to viruses in (A). The graph represents average titers (n = 3) with error bars representing the standard error of the mean (SEM). Statistical significance was calculated by Ordinary one-way ANOVA using GraphPad PRISM 7 software at a 95% confidence interval. Relative significance is indicated by asterisks (p<0.0001 = ****). (D) Sequence alignment of flavivirus C α3 helices with the residue of interest N67 denoted by an arrow above. Sequence accession numbers [ZIKV MR766: AMR39835.1, ZIKV P6740: AVK43549.1, ZIKV PRV: AMC13911.1, ZIKV PF13: ARB08112.1, ZIKV Senegal: AMR39832.1, DENV 1: ADK37471.1, DENV 2: NP_056776.2, DENV 3: ABW82020.1, DENV 4: ARM59249.1, WNV: Q9Q6P4.2, JEV: NP_059434.1, YFV: NP_041726.1, SLEV: YP_001008348.1, MVEV: NP_051124.1, DTV: AAL32169.1, POWV: NP_620099.1, TBEV: ABI 31771.1, LGTV: QBR53298.1].

The primary role of C protein in flavivirus biogenesis is genome packaging. Of the four α helices that comprise the C protein (α1–4), the predicted functions for α1, α2, and α4 include lipid binding, dimerization, and RNA binding, respectively [35,39,40]. In contrast, the role of the α3 helix remains poorly understood. Here, we investigated the functional role of α3 in mediating virus assembly and virion morphogenesis by mutational and biochemical analyses. Alanine substitutions were introduced by site-directed mutagenesis on ZIKV containing a fluorescently tagged NS2A (ZIKV_venus_), which enabled us to measure virus spread by fluorescence microscopy (Fig 1A). We selected C protein residues I66, N67, R68, and S71 from the α3 for mutagenesis based on the side chain orientation and surface exposure from the ZIKV C crystal structure (PDB:5YGH, Fig 1B) and NMR structure (PDB:6C44). We generated a quadruple alanine substitution [_66_INRWGS_71_/_66_AAAWGA_71_ (ZIKV_venus_-Cm)] as well as individual alanine substitutions ZIKV_venus_-C_I66A_, ZIKV_venus_-C_N67A_, ZIKV_venus_-C_R68A_, and ZIKV_venus_-C_S71A_. Individual alanine substitution sequences were analyzed by AlphaFold, which predicted structures similar to WT ZIKV C for all four mutants (S1 Fig) [51]. The wild-type and mutant ZIKV_venus_ clones were transfected into HEK 293-T cells, and cell culture supernatants were collected after 4 days. We infected Vero E6 cells with the collected supernatants to visualize phenotypic differences in virus spread based on fluorescent cell cluster sizes at 5 days post infection (d.p.i). (Fig 1A). In cells infected with ZIKV_venus_, we observed large foci containing >200 fluorescent cells. In contrast, the ZIKV_venus_-C_I66A_, ZIKV_venus_-C_N67A_, and ZIKV_venus_-C_R68A_ mutant viruses formed very few, small fluorescent foci containing approximately 10–20 cells, and the ZIKV_venus_-C_S71A_ mutant virus formed large fluorescent clusters of >100 cells (Fig 1A). The ZIKV_venus_-Cm mutant was severely attenuated, with very few cells expressing green fluorescence and no observable spread from cell to cell. We further estimated the virus titer from the supernatants of transfected cells by plaque assay in Vero E6 cells (Fig 1C). Wild-type ZIKV_venus_ had an average titer of 3.20×10^5^ PFU/ml, and the ZIKV_venus_-C_S71A_ mutant virus was ~2 log lower with an average titer of 2.93×10^3^ PFU/ml. In contrast, the virus mutants ZIKV_venus_-C_I66A_, ZIKV_venus_-C_N67A_, and ZIKV_venus_-C_R68A_ did not form observable plaques, suggesting that these three residues on α3 are essential for virus production. ZIKV_venus_-Cm was also non-plaque forming. In the structure of ZIKV C, N67 appears significantly more solvent accessible than I66 and R68 (Fig 1B); therefore, we selected C_N67_ as our residue of interest for further studies. Intriguingly, even though C_N67_ residue is conserved among different strains of ZIKV, it is less conserved among other *Aedes*-transmitted flaviviruses when compared to C_I66_ and C_R68_ residues (Fig 1D).

### N67A mutation of ZIKV C does not affect oligomerization or RNA binding *in vitro*

**Fig 2:**
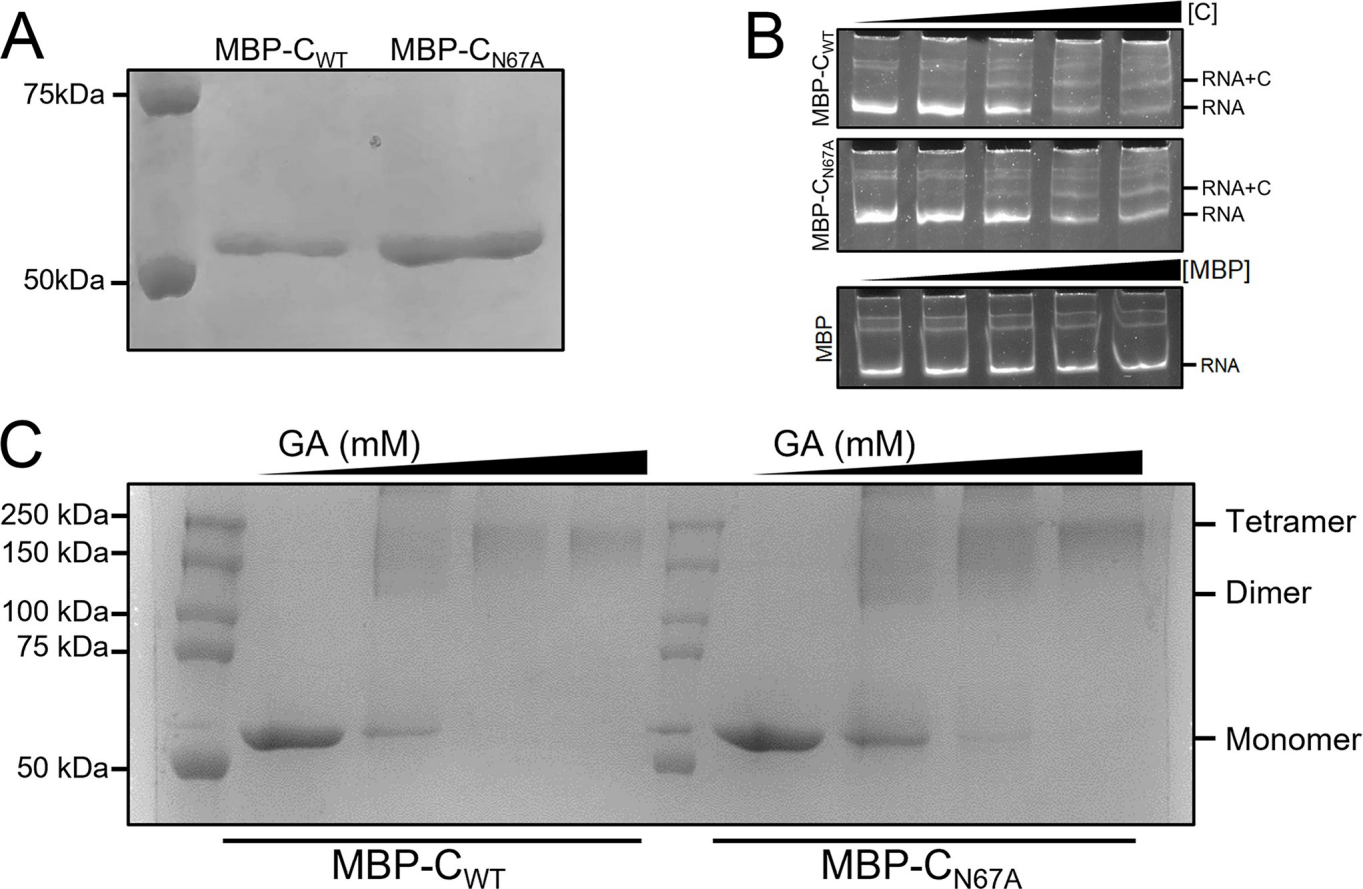
Biochemical characterization of WT and N67A mutant C proteins. Wild-type (MBP-C_WT_) and N67A mutant (MBP-C_N67A_) C were expressed and purified as MBP fusion proteins. (A) SDS-PAGE gel showing the purified MBP-C_WT_ and MBP-C_N67A_. (B) EMSA showing binding of MBP-C_WT_ (top panel), MBP-C_N67A_ (middle panel), or MBP alone (bottom panel) to RNA representing 5’UTR and C gene of ZIKV. Relative molar ratios of RNA: protein are indicated by the triangle above, from left to right: 1:0, 1:1.5, 1:3, 1:6, 1:9. (C) SDS-PAGE of samples from glutaraldehyde (GA) crosslinking assay. Samples from MBP-C_WT_ and MBP-C_N67A_ after 20 min crosslinking reaction. Relative GA concentrations are indicated by triangles above, from left to right: 0 mM, 0.25 mM, 0.5 mM, 1.0 mM. The size of the C-MBP monomer, dimer, and possible tetramer is indicated on the right.

To test whether attenuation of the ZIKV_venus_-C_N67A_ mutant virus was due to C protein defects that cause inability to fold properly, dimerize, or bind nucleic acids, we expressed wild-type (MBP-C_WT_) and N67A mutant (MBP-C_N67A_) C proteins as N-terminal MBP fusion proteins in bacteria which were then purified by affinity chromatography using an amylose resin followed by Hi-Trap Heparin column. Both MBP-C_WT_ and MBP-C_N67A_ proteins were stable and purified to homogeneity as determined by Size Exclusion Chromatography (S2 Fig), with a molecular weight of 54 kDa as determined by SDS-PAGE (Fig 2A). We then evaluated whether the C_N67A_ mutation influenced capsid-RNA binding *in vitro* by performing an Electrophoretic Mobility Shift Assay (EMSA) (Fig 2B). Both MBP-C_WT_ and MBP-C_N67A_ proteins showed a concentration-dependent binding to RNA representing the 5’UTR and C gene of ZIKV; we observed a shift in the RNA band towards a higher molecular weight RNA+C protein complex in correlation with an increasing ratio of protein to RNA. We next tested if the C_N67A_ mutation affects the oligomerization of C protein by glutaraldehyde (GA) crosslinking (Fig 2C). At a lower concentration of GA (0.25 mM), both MBP-C_WT_ and MBP-C_N67A_ formed dimer (>100 kDa) as well as higher order oligomers, possibly tetramers. At higher concentrations of GA (0.5 and 1.0 mM), both proteins are oligomerized to possible tetramers (>200 kDa). These data indicate that MBP-C_WT_ and MBP-C_N67A_ have similar oligomerization properties (Fig 2C). Taken together, our results prove that the C_N67A_ mutation does not affect the RNA binding and oligomerization properties of the C protein.

### N67A mutation does not alter the intracellular distribution of ZIKV C protein in mammalian cells

**Fig 3:**
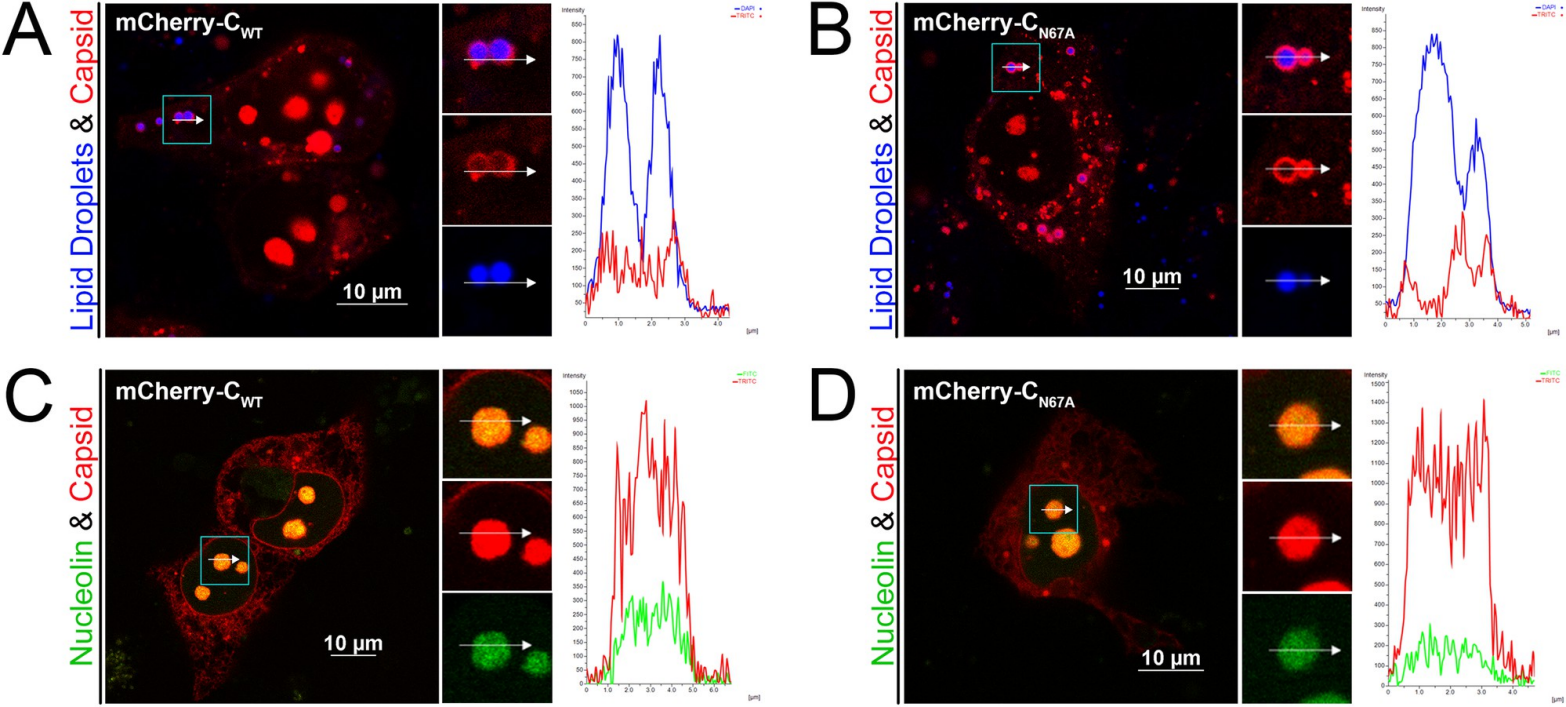
Colocalization of WT and N67A mutant C protein to lipid droplets and nucleolus. N-terminal mCherry tagged WT (mCherry-C_WT_) and N67A mutant (mCherry-C_N67A_) C proteins were expressed in JEG-3 cells, and their colocalization to LD and nucleolus were determined by confocal microscopy. (A-B) Fluorescent micrographs of JEG-3 cells transfected with mCherry-C_WT_ (A) or mCherry-C_N67A_ (B) and stained with MDH for LD (blue). (C-D) Fluorescent micrographs of JEG-3 cells co-expressing mCherry-C_WT_ (C) or mCherry-C_N67A_ (D) with nucleolar maker GFP-nucleolin. Regions of interest (ROI) are marked and represented as zoomed images to the right in separate channels. Line graphs to the right of each image show the relative intensity of red and blue (A-B) or red and green (C-D) channels corresponding to the white arrow inside the ROI. All images were acquired at 24 h.p.t.

In flavivirus-infected cells, C protein localizes to the ER and lipid droplets (LD) with functional implications for virus assembly [39,40]. The C protein also localizes to the nucleolus, although the importance of this phenomenon is not yet understood [40,41]. We tested whether C_N67A_ affects the localization of C to LD or the nucleolus by ectopic expression of N-terminal mCherry tagged C proteins (mCherry-C_WT_ and mCherry-C_N67A_) in JEG-3 cells followed by live confocal microscopy. In cells stained with MDH for LD visualization, mCherry-C_WT_ (Fig 3A) and mCherry-C_N67A_ (Fig 3B) were observed localizing to the surface of LD, suggesting that both C proteins interact with the LD membrane. We then co-expressed mCherry-tagged C protein with GFP tagged nucleolin, a nucleolar marker, in JEG-3 cells. We observed clear colocalization of both mCherry-C_WT_ (Fig 3C) and mCherry-C_N67A_ (Fig 3D) with GFP-nucleolin.

### C_N67A_ mutant virus has a significant defect in ZIKV assembly

**Fig 4:**
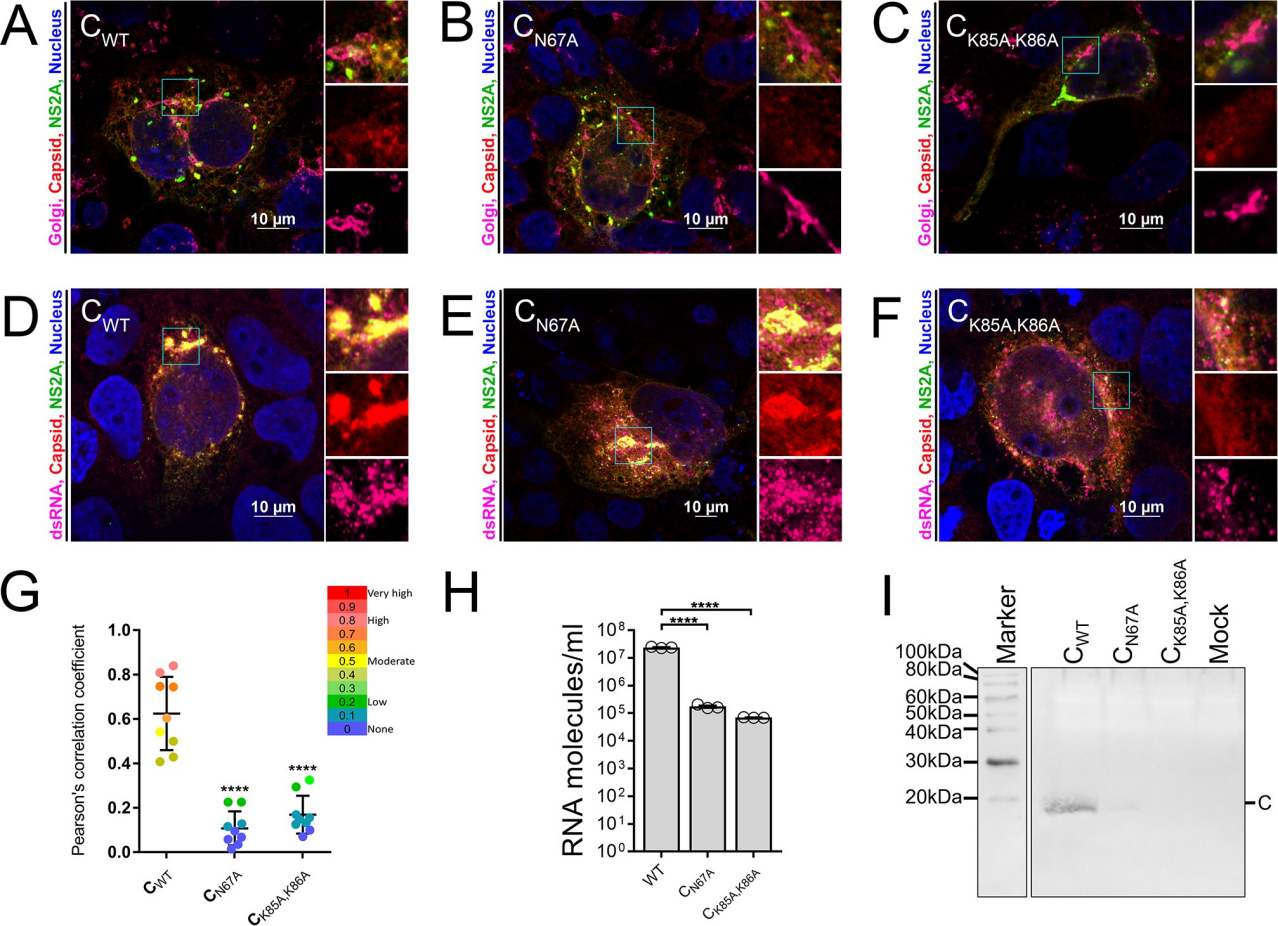
Effect of C N67A mutation on ZIKV assembly and replication. JEG-3 cells were transfected with full-length cDNA clones of WT ZIKV_venus_ (A, D) ZIKV_venus_-C_N67A_ (B, E) or C assembly negative control mutant K85A/K86A (ZIKV_venus_-C_K85A/K86A_) (C, F). Permeabilized cells were probed with antibodies against C (red) and Golgi marker Giantin (magenta) to determine the localization of C protein to the Golgi apparatus (A-C) or anti-dsRNA antibody (magenta) to detect replicating viral RNA (D-F). All cells were stained with Hoechst (blue) to detect the nucleus. The green color represents venus-tagged NS2A translated from the viral RNA. ROIs are outlined in cyan and represented as zoomed images to the right in separate channels. (G) Pearson’s correlation coefficients were calculated for the colocalization of C protein and Golgi using Nikon NIS Elements software. ROIs containing Golgi were selected from the confocal micrographs for each virus for calculation. Data points represent Pearson’s correlation coefficient (n = 9), with error bars representing mean and standard deviation (SD). Statistical significance was calculated by Ordinary one-way ANOVA using PRISM software at a 95% confidence interval. Relative significance is indicated by asterisks (p<0.0001 = ****). The heatmap indicates the colocalization range, and the data points are colored accordingly. (H-I) Detecting release of assembled virus in culture supernatants by qRT-PCR (H) and Western blot (I) 48 h.p.i. (H) Number of RNA molecules per ml of cell culture supernatant estimated by qRT-PCR. The number of RNA molecules/ml was calculated based on the Ct standard curve generated from ZIKV genomic RNA of a known quantity. The graph represents average RNA molecules/ml (n = 3) with error bars representing SEM. Statistical significance was calculated by Ordinary one-way ANOVA using GraphPad PRISM 7 software at a 95% confidence interval. Relative significance is indicated by asterisks (p<0.0001 = ****). Graph normalized to mock infected samples. (I) Western blot represents virus pellets obtained after ultracentrifugation of the cell culture supernatants probed with anti-ZIKV C antibody. Label C on the right indicates a band corresponding to the released ZIKV C protein.

We next tested whether N67A mutation causes defects in the assembly or replication of ZIKV_venus_ in JEG-3 cells by immunofluorescence (IF) analysis for C localization to Golgi (Fig 4A–4C) and dsRNA production (Fig 4D–4F), respectively. In these experiments, we used cells transfected with plasmids encoding wild-type ZIKV_venus_, ZIKV_venus_-C_N67A,_ or an assembly deficient control ZIKV_venus_-C_K85A/K86A_ viruses, all expressing venus-NS2A protein (green) [40]. When ZIKV_venus_ transfected cells were probed with an anti-giantin antibody as a Golgi marker (magenta) and an anti-ZIKV C antibody (red), we observed C protein localizing to ER and Golgi (Fig 4A). In ZIKV_venus_-C_N67A_ and ZIKV_venus_-C_K85A/K86A_ transfected cells, C protein is found localized to the ER but less on the Golgi compared to the wild-type C protein (Fig 4B and 4C). Pearson’s correlation coefficient analysis of ZIKV_venus_ infected cells showed an average value of 0.625±0.147 for the colocalization of C protein with Golgi (Fig 4G). In contrast, cells transfected with ZIKV_venus_-C_N67A_ showed an average value of 0.108±0.068 suggesting a significantly reduced colocalization of C protein with Golgi, much like the assembly deficient control ZIKV_venus_-C_K85A/K86A_ which showed an average value of 0.169±0.076. To determine if the inability to assemble is a byproduct of a replication defect, we probed ZIKV_venus_, ZIKV_venus_-C_N67A_, or ZIKV_venus_-C_K85A/K86A_ transfected cells for the presence of dsRNA using dsRNA specific antibody (Fig 4D–4F). Distinct dsRNA-associated foci (magenta) were observed throughout the cells in all samples, confirming that replication is unaffected by mutation of C_N67A_ or C_K85A/K86A_. The assembly defect of ZIKV_venus_-C_N67A,_ as inferred from the IF analysis, was further verified by qRT-PCR and western blot to evaluate viral genomic RNA and virus particles released from cells, respectively. Intact virus particles released from cells were harvested from the cell culture supernatants by ultracentrifugation using a sucrose cushion, and RNA molecules were quantified by qRT-PCR (Fig 4H). In wild-type ZIKV_venus_ samples, we detected an average of 2.37×10^7^ RNA molecules/ml. The number of molecules detected in ZIKV_venus_-C_N67A_ samples was ~2 log lower at an average of 1.74×10^5^ RNA molecules/ml, similar to the assembly deficient control ZIKV_venus_-C_K85A/K86A,_ which gave an average value of 6.69×10^4^ RNA molecules/ml. A second aliquot of the purified virus pellet obtained by ultracentrifugation was used to detect the presence of C protein via western blot using an anti-ZIKV C antibody (Fig 4I). A band corresponding to the cleaved C monomer at <20 kDa was observed for ZIKV_venus_. In comparison, no detectable C band was observed in ZIKV_venus_-C_N67A,_ similar to the negative controls purified from ZIKV_venus_-C_K85A/K86A_ and mock-infected cells.

### ZIKV C tolerates a positively charged residue at position 67 but not hydrophobic residues

**Fig 5:**
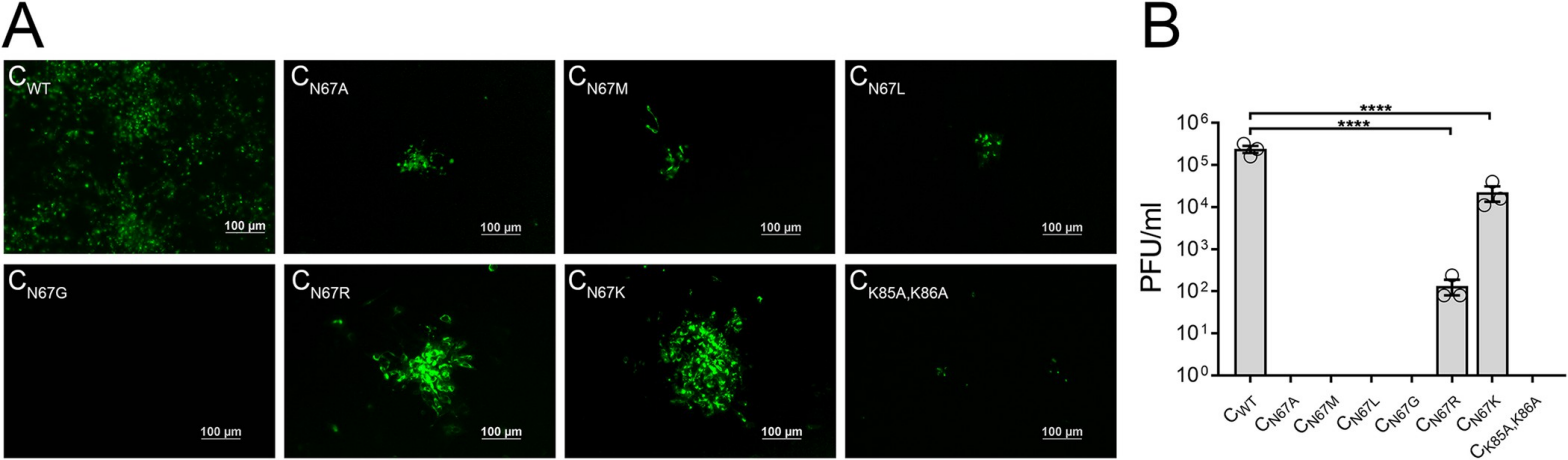
Amino acid substitution of C residue 67. (A) Micrographs showing fluorescent clusters of Vero E6 cells infected with WT ZIKV_venus_ or C mutant viruses ZIKV_venus_-C_N67A_, N67M(ZIKV_venus_-C_N67M_), N67G(ZIKV_venus_-C_N67G_), N67L(ZIKV_venus_-C_N67L_), N67R(ZIKV_venus_-C_R67R_), N67K(ZIKV_venus_-C_R67K_), and negative control mutant ZIKV_venus_-C_K85A/K86A_, demonstrating relative virus spread. Images were acquired at 5 d.p.i. (B) Virus titers of wild-type and mutant ZIKV_venus_ as determined by plaque assay in Vero E6 cells. The graph represents average titers (n = 3) with error bars representing SEM. Statistical significance was calculated by Ordinary one-way ANOVA using GraphPad PRISM 7 software at a 95% confidence interval. Relative significance is indicated by asterisks (p<0.0001 = ****).

To test the importance of asparagine residue at the 67^th^ position of C protein, which is conserved in all ZIKV strains but not in other flaviviruses, we replaced the N67 with G and K based on mosquito-borne flaviviruses JEV and DENV-2 sequences of C protein, and to M and R based on tick-borne flaviviruses DTV and POWV C protein sequences. We also substituted the hydrophilic N67 with a hydrophobic amino acid L. Cell culture supernatants of ZIKV_venus_ with the introduced mutations were harvested and used to infect Vero E6 cells. After 5 days, cells were analyzed by fluorescence microscopy for the formation of fluorescent cell clusters representing virus spread (Fig 5A). Wild-type ZIKV_venus_ formed large clusters of >200 fluorescent cells, whereas ZIKV_venus_-C_N67A,_ ZIKV_venus_-C_N67M,_ and ZIKV_venus_-C_N67L_ mutant viruses all formed small foci containing 10–20 fluorescent cells. The ZIKV_venus_-C_N67G_ mutant virus did not form observable fluorescent cell clusters. ZIKV_venus_-C_N67R_ formed clusters with 30–50 cells, and ZIKV_venus_-C_N67K_ formed large clusters with an average of ~100 cells. The ZIKV_venus_-C_K85A/K86A_ assembly mutant was used as a negative control for this assay, which showed highly attenuated cluster formation. Cell culture supernatants were also analyzed to determine virus titer by plaque assay (Fig 5B). ZIKV_venus_ had a titer of 3.20×10^5^ PFU/ml. Mutant viruses that formed very small or no clusters (ZIKV_venus_-C_N67A,_ ZIKV_venus_-C_N67M,_ ZIKV_venus_-C_N67L,_ and ZIKV_venus_-C_N67G_) were all non-plaque forming and showed no measurable titer. ZIKV_venus_-C_N67K_ had sizeable fluorescent cell clusters, and ZIKV_venus_-C_N67R,_ which showed 25–30 cell clusters, gave significantly reduced titers of 1.33×10^2^ and 2.24×10^4^ PFU/ml, respectively.

### A second site reversion in M protein rescues ZIKV-C_N67A_

**Fig 6:**
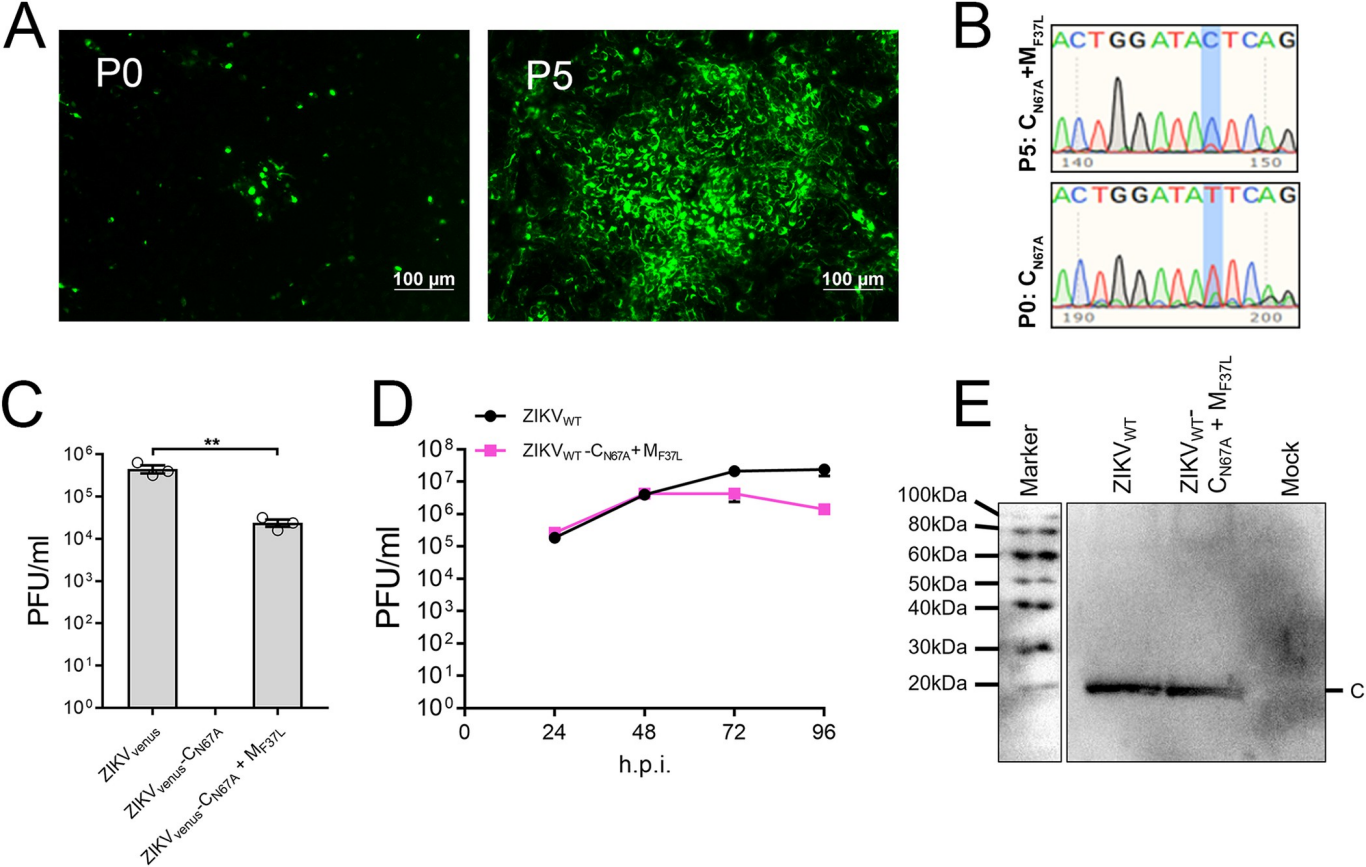
Identification and characterization of C_N67A_+M_F37L_ revertant. (A) Micrographs showing fluorescent clusters of Vero E6 cells infected with ZIKV_venus_-C_N67A_ at passage 0 (P0) and passage 5 (P5). (B) Sanger sequencing results analyzed using FinchTV software showing the region of mutation in M protein in the P5 revertant virus. A single nucleotide T→C mutation (highlighted in blue) created an F→L amino acid mutation in the M protein resulting in a ZIKV_venus_-C_N67A+_+M_F37L_ double mutant. (C) Virus titers of ZIKV_venus_-C_N67A_ mutant and ZIKV_venus_-C_N67A+_+M_F37L_ revertant compared to wild-type ZIKV_venus_ as determined by plaque assay on Vero E6 cells. The graph represents average titers (n = 3) with error bars representing SEM. Statistical significance was calculated by Ordinary one-way ANOVA using GraphPad PRISM 7 software at a 95% confidence interval. Relative significance is indicated by asterisks (p<0.01 = **). (D) Growth curve analysis of ZIKV_WT_ and ZIKV_WT_-C_N67A+_+M_F37L_. The graph represents average titers (n = 3) at 24-, 48-, 72-, and 96 h.p.i with error bars representing SEM. (E) Western blot of virus pellets obtained after ultracentrifugation of the cell culture supernatants probed with anti-ZIKV C antibody. Label -C on the right indicates a band corresponding to the released ZIKV C protein.

Even though the C_N67A_ mutant virus is severely attenuated, the mutant provided the opportunity to screen for revertants that rescue the phenotype. We used the fluorescent ZIKV_venus_-C_N67A_ virus collected from transfected HEK-293T cells (P0) to infect Vero E6 cells in triplicate. After 6 days, supernatants were collected and passaged consecutively up to five times in Vero E6 cells. Cells were monitored for the emergence of sizeable fluorescent cell clusters indicating rescued phenotype. After five passages (P5), large fluorescent groups containing >100 cells per cluster were observed in all three replicates (Fig 6A). We performed RT-PCR on the P5 supernatant to amplify the gene encoding C, followed by Sanger sequencing of the amplicons. Out of three replicates, two had reverted to wild-type ZIKV_venus_-C_N67_, and the third retained the C_N67A_ mutation. To further analyze this third replicate, we performed RT-PCR and Sanger sequencing of genes encoding prM and E structural proteins, revealing a single nucleotide second site mutation in M protein changing F37 to L37 (ZIKV_venus_-C_N67A_+M_F37L_) (Fig 6B). To confirm the rescue of phenotype by C_N67A_+M_F37L_, we compared its titer with the titers of ZIKV_venus_-C_N67A_ and wild-type ZIKV_venus_ obtained by plaque assay. While ZIKV_venus_-C_N67A_ did not form plaques, ZIKV_venus_-C_N67A_+M_F37L_ had an average titer of 2.4×10^4^ PFU/ml compared to ZIKV_venus_ which gave an average tier of 4.53×10^5^ PFU/ml (Fig 6C). Next, to confirm the M_F37L_ mutation alone rescues the attenuated phenotype of ZIKV-C_N67A_, we engineered non-fluorescent ZIKV (ZIKV_WT_) cDNA to obtain viruses containing C_N67A_ (ZIKV_WT_-C_N67A_) single mutation and C_N67A_+M_F37L_(ZIKV_WT_-C_N67A_+M_F37L_) double mutations. We then compared the growth kinetics of ZIKV_WT_-C_N67A_+M_F37L_ with ZIKV_WT_ in Vero E6 cells (Fig 6D). Cell culture supernatants had comparable virus titers at 96 h.p.i. The ZIKV_WT_-C_N67A_+M_F37L_ reached an average titer of 1.41×10^6^ PFU/ml, compared to ZIKV_WT,_ which gave an average titer of 2.42×10^7^ PFU/ml. To confirm the assembly and release of ZIKV_WT_-C_N67A_+M_F37L_ from infected cells, we purified the virus from supernatants of infected Vero E6 cells and performed western blot analysis using an anti-ZIKV C antibody. A band corresponding to ZIKV C was observed near <20 kDa in ZIKV_WT_ and double mutant ZIKV_WT_-C_N67A_+M_F37L_ samples, confirming virus release (Fig 6E).

### The amino acid at position 37 of M significantly impacts the ZIKV virus life cycle

**Fig 7:**
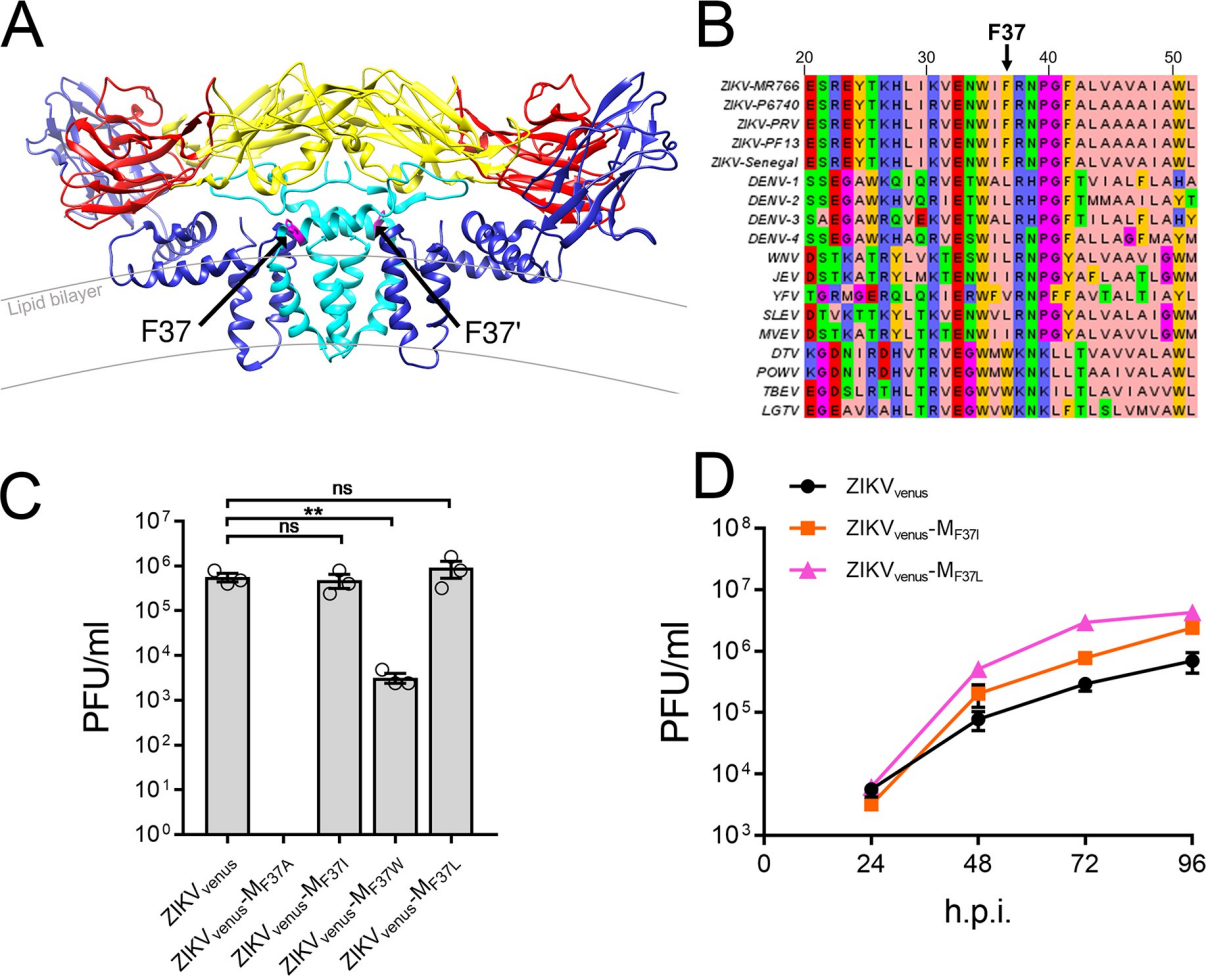
Mutational analysis of ZIKV M residue 37. (A) Cryo-EM structure of mature ZIKV M/E heterodimers (PDB:6CO8) showing the position of M_F37_ residue in magenta and marked with black arrows. E protein chains are shown with standard domain coloring (domain I: red, domain II: yellow, domain III: blue), and M protein chains are represented in cyan. The lipid bilayer is indicated by curved gray lines. (B) Multiple sequence alignment of flavivirus M perimembrane helix (M-H1) annotated in Jalview with Zappo coloring. The residue of interest F37 is denoted by an arrow above. Sequence accessions numbers [ZIKV MR766: AMR39835.1, ZIKV P6740: AVK43549.1, ZIKV PRV: AMC13911.1, ZIKV PF13: ARB08112.1, ZIKV Senegal: AMR39832.1, DENV 1: ADK37471.1, DENV 2: NP_056776.2, DENV 3: ABW82020.1, DENV 4: ARM59249.1, WNV: Q9Q6P4.2, JEV: NP_059434.1, YFV: NP_041726.1, SLEV: YP_001008348.1, MVEV: NP_051124.1, DTV: AAL32169.1, POWV: NP_620099.1, TBEV: ABI 31771.1, LGTV: QBR53298.1] (C) Virus titers (passage 0) calculated from plaque assay of ZIKV_venus_, and M_37_ substitution mutant viruses F37A(ZIKV_venus_-M_F37A_), F37I(ZIKV_venus_-M_F37I_), F37W(ZIKV_venus_-M_F37W_), and F37L(ZIKV_venus_-M_F37L_). The graph represents average titers (n = 3) with error bars representing SEM. Statistical significance was calculated by Ordinary one-way ANOVA using GraphPad PRISM 7 software at a 95% confidence interval. Relative significance is indicated by asterisks (p<0.01 = **, p>0.05 considered not significant (ns)). (D) Growth curve analysis of ZIKV_venus_, ZIKV_venus_-M_F37I_, and ZIKV_venus_-M_F37L_ viruses. The graph represents average titers (n = 3) at 24-, 48-, 72-, and 96 h.p.i with error bars representing SEM.

In the cryo-EM structure of mature ZIKV M/E heterodimer (PDB:6CO8), M_F37,_ which reverted to L and rescued the C_N67A_ mutant, is located on the first perimembrane helix of M (M-H1) near the outer leaflet of the lipid bilayer that makes up the viral membrane (Fig 7A) [10]. M_F37_ is not a highly conserved residue, although the phenylalanine is conserved within ZIKV strains, and relative hydrophobicity is conserved across flaviviruses (Fig 7B). To evaluate the significance of the residue at position 37 in ZIKV M, we generated a ZIKV_venus_-M_F37A_ mutant by site-directed mutagenesis. We also included ZIKV_venus_-M_F37I_, ZIKV_venus_-M_F37W_, and ZIKV_venus_-M_F37L_, to analyze the effect of analogous residues found in related flaviviruses JEV, DENV, and POWV, respectively. Mutations were first introduced into ZIKV_venus,_ and virus production was estimated by plaque assay of cell culture supernatants. Wild-type ZIKV_venus_ had an average titer of 5.6×10^5^ PFU/ml, whereas F37A substitution was lethal, strongly suggesting that F37 is a critical residue for viral production (Fig 7C). ZIKV_venus_-M_F37W_ was attenuated, producing a titer of 3.20×10^3^ PFU/ml. ZIKV_venus_-M_F37I_ and ZIKV_venus_-M_F37L_ resulted in high virus titers of 4.8×10^5^ PFU/ml and 9.07×10^5^ PFU/ml, respectively, showing that these mutations are tolerated in ZIKV (Fig 7C). We next compared the growth kinetics of the mutants ZIKV_venus_-M_F37L_ and ZIKV_venus_-M_F37I_ with wild-type ZIKV_venus_ (Fig 7D). ZIKV_venus_-M_F37W_ was not included in this assay due to its reduced titer. Both ZIKV_venus_-M_F37L_ and ZIKV_venus_-M_F37I_ resulted in titers that exceeded ZIKV_venus_ by 48 h.p.i. and continued to outperform ZIKV_venus_ until 96 h.p.i., reaching final titers of 4.27×10^6^ PFU/ml and 2.40×10^6^ PFU/ml respectively compared to ZIKV_venus_ that reached 6.93×10^5^ PFU/ml, indicating that M_F37L_ and similar hydrophobic residues provide optimal growth advantage for ZIKV production in the absence of any C protein mutation.

### The critical functions of C α3 and M-H1 are conserved in DENV

**Fig 8:**
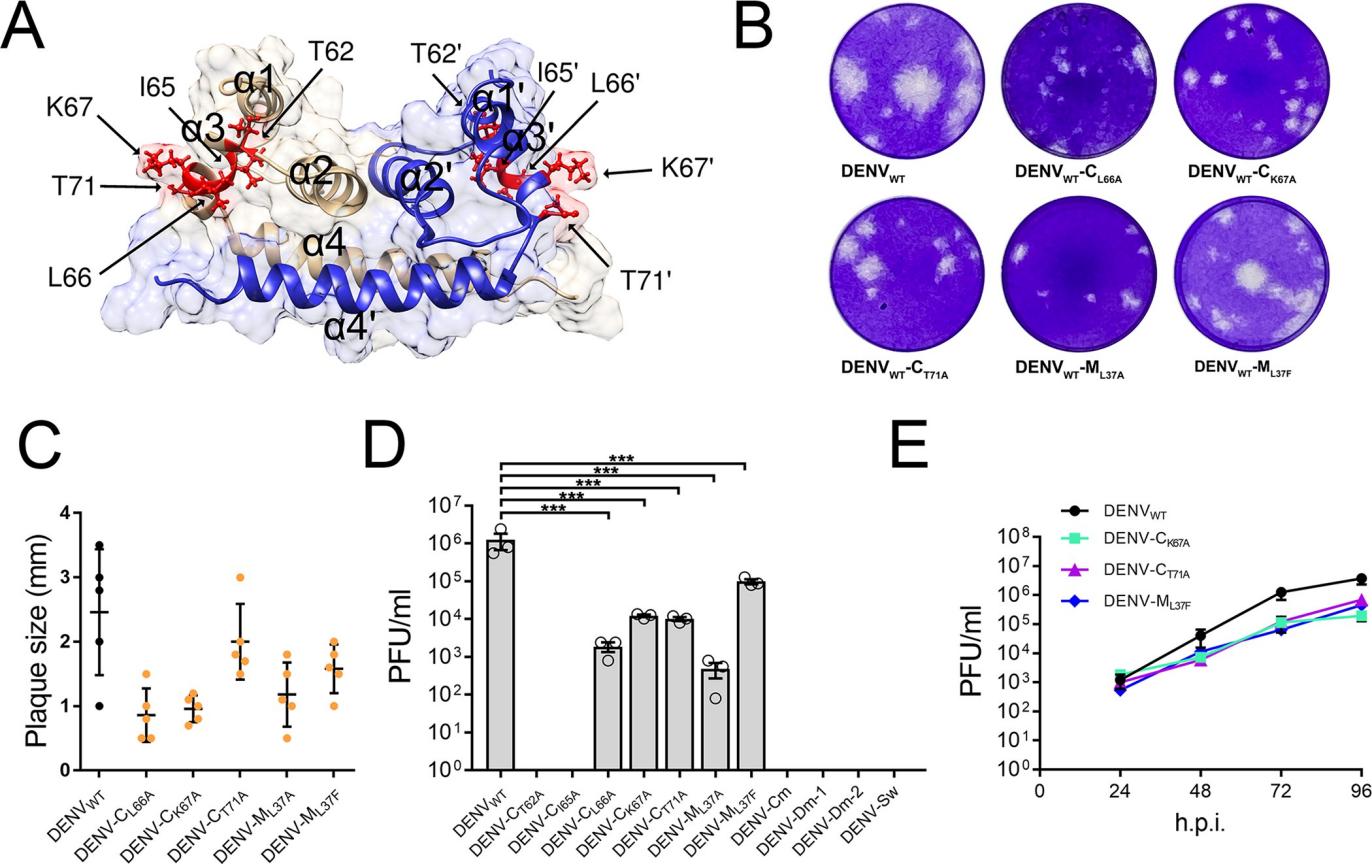
Mutational analysis of DENV C α3 and M_L37_. (A) NMR structure of DENV C (PDB:1R6R) generated using UCSF Chimera software with α3 residues selected for mutational analysis shown as ball and stick and colored in red. Chain A is shown in beige, chain B is shown in blue, and helices α1–4 are labeled on the structure. (B) Plaque morphologies of DENV_WT_ and plaque-forming C mutants L66A(DENV-C_L66A_), K67A(DENV-C_K67A_), and T71A(DENV-C_T71A_), as well as plaque-forming M mutants L37A(DENV-M_L37A_), and L37F(DENV-M_L37F_) in BHK-15 cells fixed at 7 d.p.i. and stained with crystal violet. (C) The graph shows plaque diameters measured from DENV_WT_ (black circles) or mutant DENV (orange circles) plaque assays shown in (B). The graph represents average plaque diameters (n = 5) with error bars representing SD. Statistical significance was calculated by Ordinary one-way ANOVA using GraphPad PRISM 7 software at a 95% confidence interval. Relative significance indicated by asterisks (p<0.01 = **, p<0.001 = ***, values with p>0.05 considered not significant (ns)). (D) Virus titers (passage 0) calculated from plaque assay in BHK-15 cells of wild-type and mutant DENV. DENV-Cm represents a combination mutant (C-_62_TAGILKRWGT_71_/_62_AAGAAARWGA_71_), DENV-Dm-1 represents a double mutant one (C-_66_LK_67_/_66_AA_67_), DENV-Dm-2 represents a double mutant two (C-_67_KRWGT_71_/_67_ARWGA_71_), and DENV-Sw represents helix swap mutant with ZIKV C α3 in place of DENV C α3 (C-_62_TAGILKRWGT_71_/_62_SLGLINRWGSVI_73_). The graph represents average titers (n = 3) with error bars representing SEM. Statistical significance was calculated by Ordinary one-way ANOVA using GraphPad PRISM 7 software at a 95% confidence interval. Relative significance is indicated by asterisks (p<0.001 = ***). (E) Growth curve analysis of WT DENV, DENV-C_K67A_, DENV-C_T71A,_ and DENV-M_F37L_ viruses (passage 0) in BHK-15 cells infected at MOI = 0.01. The graph represents average titers (n = 3) at 24-, 48-, 72-, and 96 h.p.i. with error bars representing SEM.

We next evaluated whether residues analogous to C α3 and M_F37_ of ZIKV are necessary for assembly in DENV, a closely related mosquito-borne flavivirus. Based on the NMR structure of DENV C protein (PDB:1R6R) [42], we selected surface-exposed residues L66, K67, and T71, as well as internally facing residues T62 and I65 on the C α3 for alanine substitution and further characterization of the mutants using DENV-2 16681 infectious cDNA clone pD2/IC-30P represented as DENV_WT_ (Fig 8A) [52]. Viruses were generated by *in vitro* transcription and electroporation, and phenotypes were evaluated by plaque assays of the supernatant using BHK-15 cells (Fig 8B–8D). DENV_WT_ formed plaques with an average diameter of 2.5±0.98 mm (Fig 8B and 8C) and resulted in a titer of 1.25×10^6^ PFU/ml (Fig 8D). Compared to DENV_WT_, the mutant viruses DENV-C_L66A_ and DENV-C_K67A_ produced significantly smaller plaques with an average diameter of 0.9±-.42 and 1.0±0.21 mm, and reduced titers, 1.87×10^3^ and 1.23×10^4^ PFU/ml respectively. DENV-C_T71A_ produced large plaque sizes with an average diameter of 2.0±0.59 mm, giving a 1.01×10^4^ PFU/ml titer. Internally facing residue mutants DENV-C_T62A_ and DENV-C_I65A_ were non-plaque forming, likely due to compromised tertiary interactions at the C protein dimer interface. Additionally, we generated double mutants combining alanine substitutions of externally oriented residues [_66_LK_67_/_66_AA_67_ (DENV-Dm-1)] or [_67_KRWGT_71_/_67_ARWGA_71_ (DENV-Dm-2)], neither of which formed observable plaques. A multiple alanine substitution mutation [_62_TAGILKRWGT_71_/_62_AAGAAARWGA_71_ (DENV-Cm)] targeting the non-conserved and surface-exposed residues was also lethal. Finally, to determine whether the overall sequence specificity of α3 is significant, we generated a full α3 helix swap with DENV expressing the α3 sequence of ZIKV [(DENV-_62_TAGILKRWGT_71_/_62_SLGLINRWGSVI_71_) (DENV-Sw)] which was also non-plaque forming.

The residue analogous to ZIKV M_F37_ is leucine in DENV (Fig 7B). To determine the importance of this residue in DENV, we first generated a DENV-M_L37A_ mutant which was attenuated, produced small plaques (1.2±0.50 mm diameter), and formed an average titer of 4.80×10^2^ PFU/ml compared to DENV_WT_ (2.5±0.98 mm diameter plaques) with a titer of 1.25×10^6^ PFU/ml (Fig 8B–8D)_._ When we substituted M_L37_ with the analogous residue from ZIKV to generate DENV-M_L37F_, the mutant virus was less attenuated, with 1.6±0.38 mm diameter plaques and a titer of 9.87x10^4^ PFU/ml (Fig 8B–8D). We then compared the growth kinetics of DENV-C_K67A_, DENV-C_T71A_, and DENV-M_L37F_ with DENV_WT_ in BHK-15 cells. Mutants with titers <1.0×10^4^ PFU/ml were not included in this assay. The growth rate of the DENV mutants was consistently lower than DENV_WT_ (Fig 8E). The final average titers after 96 h.p.i. of DENV-C_K67A_, DENV-C_T71A_, and DENV-M_L37F_ were 1.92×10^5^, 6.93×10^5^, and 4.53×10^5^ PFU/ml, respectively, compared to DENV_WT_ which reached an average titer of 3.73×10^6^ PFU/ml.

## Discussion

The flavivirus C protein mediates viral RNA genome packaging and nucleocapsid budding into the ER lumen to form immature viral particles. We have identified a novel role for the α3 helix of a, *Aedes*-transmitted flavivirus C protein in orchestrating virion assembly through molecular genetics and biochemical analyses. While several studies have explored the function of C protein residues, few have attributed key roles for amino acids in the relatively short α3 helix. Based on available crystal structures of mosquito-borne flavivirus C proteins, surface exposed on C α3 is a common feature despite the non-conserved sequence (S3 Fig). JEV α3 mutations C_K63_ and C_L66_ to alanine resulted in mild impairment of virus production but were not lethal [40]. However, in West Nile virus, large truncations removing the entire α2 and α3 in a two-component recombinant virus system were tolerated, indicating that the importance of this region varies by virus [53]. Notably, most of these studies used virus-like particles, replicons, or similar systems that indirectly measure viral functions. Here, by using full-length infectious cDNA clones of ZIKV and DENV for mutational analyses, we have identified homologous functions for surface exposed α3 residues in mediating flavivirus assembly and uncovered a genetic interaction with M protein that can only be detected using full-length cDNA clones generating infectious virus.

In many plus-strand RNA viruses, capsid protein binding to an RNA molecule is the switch that triggers nucleocapsid core formation via lateral interactions with nearby capsid proteins [54–57]. In alphaviruses, the capsid nucleation forms core-like particles in the absence of other structural proteins, requiring only a short nonspecific oligomer for initiation [58]. SARS-CoV-2 has been proposed to utilize intracellular liquid-liquid phase separation mechanisms in conjunction with a packaging signal for assembly [59]. Although the RNA binding regions of C in flaviviruses have been identified, the nucleation process and lateral interactions between C dimers required for nucleocapsid formation are still undetermined. Lethal mutations such as C_K31A,R32A_ and C_K85A/K86A_ have been shown to prevent assembly by indirectly interfering with genome packaging; however, these mutations have not been associated with the incorporation of nucleocapsid core into the immature virus particle [40]. Thus far, our study is the first to identify a specific residue of C protein that inhibits assembly without affecting the RNA binding or destabilizing the protein structure, thereby affecting capsid protein dimerization. The position of the α3 helix in the flavivirus C protein structure suggests that this alpha helix is an ideal candidate for lateral interactions between adjacent C proteins. Inter-dimer interactions may be critical during assembly; however, α3 has been overlooked in mutagenesis studies, presumably due to the lack of sequence conservation in this region. We have shown that alanine substitution of a single surface exposed asparagine C_N67_ in the α3 helix of ZIKV abrogates virus assembly, causing a lethal phenotype (Fig 1). The polar side chain of C_N67_ has the potential to form hydrogen-bonding interactions with nearby amino acids. Additionally, asparagine is known to form isopeptide bonds with lysine residues in unstructured protein regions during the capsid formation in bacteriophages such as HK97 [60]. Therefore, it is feasible for the solvent-exposed residue C_N67_ to be involved in an interaction with nearby C protein molecules via one of the positively charged residues in the long N terminal unstructured region such as K2, K5, K6, K7, K18, or K31, stabilizing the internal nucleocapsid core observed in the cryo-EM structures of immature ZIKV. Indeed, in the dimeric form of flavivirus C proteins, the α3 from each monomer is oriented perpendicular to the proposed RNA binding regions: α1 at the top and α4 at the bottom of the dimer. Together, the structural orientation and biochemical properties of C_N67_ presumably contribute to the lateral interactions between C proteins without obstructing the RNA packaging function. Our *in vitro* assays (Fig 2) indicate that C_N67A_ mutation does not lead to defects associated with protein folding, oligomerization, or RNA genome packaging and, therefore is presumably involved in another critical function required for virus assembly in infected cells.

During flavivirus infection, the C protein is primarily localized to the ER membrane, where it is co-translationally translocated and later in the secretory pathway as a structural component of the budded virus. However, after the NS2B/NS3 protease cleavage, the C protein has also been shown to localize to lipid droplets and the nucleus, specifically to the nucleoli in the infected cells [12,14,39]. Although the functional roles of C protein at these distinct subcellular locations remain unclear, it has been proposed that the lipid droplet binding sequesters the cleaved C protein away from assembly sites to prevent premature RNA genome packaging, whereas the functional role of C protein localizing to the nucleolus is not clearly understood [39]. In DENV, the membrane association of C protein has been linked to residues in a hydrophobic cleft, which appears to partially expose α2 based on NMR structure [39]. However, in the ZIKV dimer, the hydrophobic cleft is obstructed by α1 and the N-terminal disordered region from each C protein monomer, indicating that a different area of C protein likely mediates the membrane binding. Here, we tested the effect of C_N67A_ mutation in C protein membrane association and subcellular localization using live cell imaging. We show that the subcellular localization of C_N67A,_ specifically the colocalization with lipid droplets (Fig 3A and 3B) and the nucleoli (Fig 3C and 3D), are identical to wild-type C protein, confirming that the C protein membrane association is not dependent on residue C_N67_.

The most likely stages of the flavivirus life cycle to be impacted by the perturbation of C protein are genome packaging and virus assembly because both processes rely on direct C protein interactions. Newly assembled immature flavivirus particles that bud into the ER lumen traffic through the Golgi apparatus, where virus maturation mediated by the furin cleavage of prM protein occurs before the mature virus exits the infected cell. As such, assembly defects can be detected from the lack of colocalization of C protein with the Golgi marker Giantin [29]. Immunofluorescence showed that ZIKV containing the C_N67A_ mutation is not efficiently colocalizing with Golgi, while dsRNA formation is unaffected, implicating defects in virus assembly (Fig 4A–4F). Further testing of the C_N67A_ mutant by qRT-PCR (Fig 4H) and western blot analysis (Fig 4I) demonstrated a significant reduction in the release of viral RNA and C protein, respectively, suggesting that the defect caused must be before virus particle release. Based on these results, we conclude that C_N67A_ is an assembly-specific mutation and is the first to our knowledge that prevents assembly via a single C residue.

We next performed amino acid substitutions based on multiple sequence alignment of flavivirus C proteins (Fig 1C) to test the required biochemical properties of site C_67_ (Fig 5A and 5B). We hypothesized that the size and polarity of asparagine are essential factors in its function, which was confirmed by ZIKV_venus_ mutants in which hydrophobic residue substitutions C_N67A_, C_N67G_, and C_N67L_ resulted in non-plaque forming virus, whereas charged or polar residues C_N67R_ and C_N67K_ were tolerated and sustained plaque formation. Interestingly, the residue akin to ZIKV C_67_ in DENV is lysine, indicating a common function on C α3 despite the lack of sequence conservation. The functional role of C_N67_ likely relies on an electrostatic interaction not supported by hydrophobic residues. It requires a certain level of electron density that is met by asparagine and lysine but only partially sustained by arginine. Additionally, crystal structures show stabilizing interactions of C α3 with the backbone carbonyl oxygen of nearby residues. For example, there is an interaction between N64 and S71 in ZIKV (PDB:5YGH) as well as an interaction between D66 and R62 in WNV (PDB: 1SFK) [43,44]. The possibility that these interactions are critical and that C_67_ can only tolerate polar or charged residues is also supported by our data. Overall, these data suggest that the identity of the residue at position C_67_ plays a significant role in ZIKV assembly.

Our data also show the assembly defects of ZIKV_venus_-C_N67A_ are restored by a second site M_F37L_ mutation on the M structural protein, obtained via natural reversion or site-directed mutagenesis (Fig 6A–6D). Since the defects caused by C_N67A_ were assembly-specific, we tested whether the assembly is restored in the C_N67A_+M_F37L_ double mutant, and western blot of purified virus from infected cell supernatants confirmed the release of virus particles comparable to the wild-type levels (Fig 6E). It must be noted that the structural positioning of C_N67_ and M_F37_ places them on opposite sides of the viral lipid bilayer, making direct contact between these two residues impossible. Thus, our data provide new evidence for a genetic interaction between C and M proteins directly related to virus assembly. Our data connect several recent studies that have implicated M protein involvement in virus assembly. Mutational analysis of conserved residues on the peri-membrane helix (M-H1) of M in DENV showed that alanine substitution of four residues at the C terminal end (M_E33_, M_W35_, M_L37_ M_R38_) has a significant reduction in particle assembly [61]. Although the study did not reveal any connection to capsid protein, it implicates the direct analog M_F37_ of ZIKV identified in our revertant screen. Another study on JEV using virus-like particles that contained only prM and E pointed to M_I36F_ as an assembly mutation. However, the host-specific defect did not impair assembly in C636 mosquito cells. Since there is no C in the virus-like particle system, this study did not consider the potential interaction of M and C, which can only be observed when all structural proteins are present in an infectious virus [62]. A comparison of the virulence of the ZIKV NIID123 strain from South Asia and PRVABC59 from the Americas has identified a similar F to L reversion at amino acid 53 on M [63]. M_F53L_ mutation increased the virulence of the NIID123 strain, whereas the reciprocal M_L53F_ mutation in PRVABC59 had a negative effect. The substitution of F and L residues is strikingly similar to the M_F37L_ reported in the current study (Fig 7), where the ZIKV_venus_-M_F37L_ had a significantly increased titer, whereas the reciprocal M_L37F_ substitution in DENV reduced virus titer (Fig 8). Notably in the cryo-EM structure of immature ZIKV (PDB: 6LNU), M_F37_ appears near E protein residue 407, which is a highly conserved isoleucine in flaviviruses.

**Fig 9:**
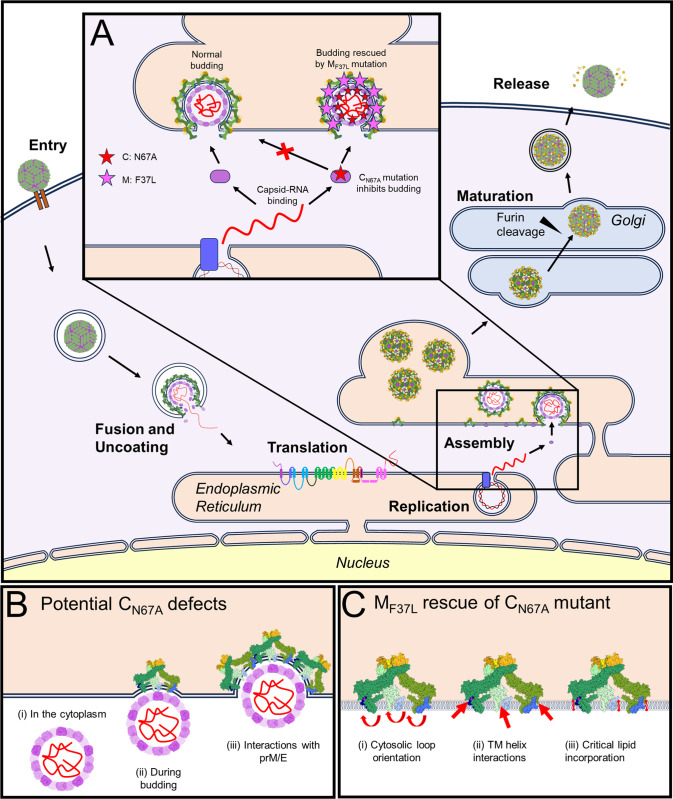
Proposed impacts of C_N67A_ and C_N67A_+M_F37L_ mutations on flavivirus assembly. (A) In the wild-type virus, capsid protein (purple) and prM/E heterodimers (green and yellow) assemble along with viral RNA (red) and the lipid bilayer to generate immature viral particles (left). Mutation C_N67A_ (red star) prevents virus assembly, leading to a lack of immature particle budding. In C_N67A_-M_F37L_ revertant (magenta star), assembly is restored (right). (B) Potential core formation defects caused by C_N67A_ mutation. (C) Potential mechanisms of M_F37L_ rescue of C_N67A_ mutant. Icons for flavivirus images were generated manually using PowerPoint and InkScape drawing tools with published cryo-EM structures of mature (PDB: 6CO8) and immature (PDB:5U4W) ZIKV structures generated using ChimeraX as templates. All icons in A are drawn using Microsoft PowerPoint. Icons representing protein structures in B and C were generated using ChimeraX https://www.rbvi.ucsf.edu/chimerax.

Supported by previous studies, our experiments provide evidence for the critical importance of M protein in flavivirus assembly. To our knowledge, this is the first evidence of a genetic interaction between C and M in flaviviruses that is required to form infectious viral particles. Recent structural studies on flaviviruses, including Binjari, Usutu, and Spondweni viruses, have shown that the orientation of the internal membrane loop (M_51_-_57_, ZIKV numbering) changes between immature and mature structures [16,17,23]. Based on our data, we propose that the internal orientation of M_51_-_57_ facilitates interaction with C protein that is allosterically regulated by M-H1 residues. Although the immature ZIKV structure has been resolved to a moderate 9 Å resolution [27,31], it is conceivable that the hydrophobic residue at position 37 in M-H1 could interact with other amino acids or the viral lipid bilayer in a specific way that promotes virus assembly (Fig 9C). Notably, recent immature virus structures are obtained by selecting immature particles that underwent incomplete maturation during normal egress from the cell; as such, they have been exposed to the low pH environment that typically triggers furin cleavage. In contrast, structures obtained from NH_4_Cl-treated neutral pH environments have not been exposed to maturation and low pH conditions and are more representative of nascent immature particle structures in the ER lumen [28]. In these immature virus structures, a density corresponding to the C protein is observed below the membrane, making direct contact with the internal portions of M and E proteins. Based on our results, we propose that M and C proteins coordinate the assembly of the immature particle, and that the interaction is lost due to the maturation-mediated glycoprotein rearrangement in the secretory pathway; therefore, the interaction is not observed in high-resolution cryo-EM structures of mature virions.

Finally, we validated the functional role of the C α3 helix in regulating virus assembly across *Aedes*-transmitted flaviviruses by performing a mutational analysis of DENV. Multiple alanine substitutions of five surface exposed side chains of α3 helix _62_TAGILKRWGT_71_/_62_AAGAAARWGA_71_ (DENV-Cm), were lethal (Fig 8A and 8D), confirming the significance of the α3 helix. The alanine substitution of C_T62_ and C_I65_ residues with internally facing side chains was lethal because the interactions are likely crucial for C protein folding and dimerization (Fig 8A–8D). Unlike ZIKV, the mutation of a single surface exposed residue did not completely abrogate virus assembly in DENV. The two amino acid mutations, C_L66A_ and C_K67A,_ resulted in 3-log and 2-log reductions in virus titer, respectively, with smaller plaque phenotypes, indicating the importance of these residues (Fig 8B and 8D). However, a double mutant combining two surface exposed residues, L66A+K67A (DENV-Dm-1) or L67A+T71A (DENV-Dm-2), resulted in a lethal phenotype (Fig 8D). Thus, we show that the functional role served by ZIKV C_N67_ is presumably orchestrated by a combination of residues L66, K67, and possibly T71 in the DENV C protein. We also created a complete helix substitution of DENV α3 helix with the α3 helix from ZIKV _62_TAGILKRWGT_71_/_62_SLGLINRWGSVI_71_ (Fig 8D, DENV-Sw), which was non-plaque forming, demonstrating the virus-specific amino acid sequence possibly required for the interactions in the function of C α3 because α3 is not a highly conserved region.

Based on the evidence presented in this study, we propose a model of *Aedes*-transmitted flavivirus assembly (Fig 9). Mutation of C_N67_ to alanine prevents assembly, indicating a critical function of the α3 helix (Fig 9A). In contrast, the second site M_F37L_ mutation rescues the assembly function, and immature viral particles can form (Fig 9A). The retention of dimerization and RNA binding activity indicates that the critical step affected by residue N67 is related to nucleocapsid core formation (Fig 9B). It is not currently known if core formation occurs prior to (i), in parallel with (ii), or during budding (iii), making it difficult to discern the defect caused by the C_N67A_ mutation. As for the M_F37_ residue, numerous possible mechanisms could be rescuing assembly, including (i) realigning the orientation of the M and E cytosolic loops to facilitate an interaction with C, (ii) creating a stabilizing interaction between the transmembrane regions of the M and E proteins that compensates for a loss of stability due to C_N67A_, or (iii) incorporation of a critical lipid moiety into the virion that aids in the assembly of immature particles (Fig 9C). Future work exploring structural differences between the C_N67A_+M_F37L_ and WT ZIKV virions will pinpoint the mechanisms involved in these crucial interactions.

There are currently no effective and approved antivirals for flaviviruses. Flavivirus assembly is a promising area for potential antiviral drug development because of the availability of near-atomic cryo-EM structures and crystal structures for all three structural proteins, C, prM, and E, crucial to producing infectious viruses. Assembly inhibitors have been effective against many viruses, including lenacapavir against HIV and DGAT1 inhibitors against HCV [64,65]. Notably, a few flavivirus assembly inhibitors are in development, including the DENV-2-specific ST148, which induces tetramerization of C protein dimers, resulting in improper incorporation of C into virions [66,67]. It is, therefore, essential to explore the functional role of the C protein in nucleocapsid formation and virus budding to pursue the development of assembly inhibitors. The evidence for the novel genetic interaction of C with M that we have obtained in this study will be instrumental in developing structure-based assembly inhibition strategies to serve as anti-flaviviral treatments.

## Materials and methods

### Viruses and cells

The DNA-launched ZIKV MR766 cDNA clones under a CMV promoter (ZIKV_WT_) and with a Venus-tagged NS2A protein (ZIKV_venus_) were used to generate wild-type and mutant ZIKV [68]. Human embryonic kidney (HEK-293T) cells were used to transfect ZIKV cDNA clones to generate virus stocks. African green monkey kidney cells (Vero E6) and Human Choriocarcinoma Cells (JEG-3) were used for plaque assays and confocal microscopy experiments, respectively. Wild-type and mutant DENV clones were generated using a cDNA clone of DENV-2 16681 (pD2/IC-30P) (DENV_WT_) under a T7 promoter [69]. *In vitro* transcribed RNA from wild-type and mutant DENV were electroporated into Baby hamster kidney cells (BHK-15). Vero E6 and HEK-293T cells were maintained in Dulbecco’s Modified Eagle’s Medium (DMEM) (Thermo Fisher Scientific, #12800–082), BHK-15 cells were maintained in Eagle’s Minimal Essential Medium (MEM) (Thermo Fisher Scientific, #41500–018), and JEG-3 cells were maintained in DMEM: Nutrient Mixture F-12 (DMEM/F-12) (Thermo Fisher Scientific, #12500–062). All media were supplemented with 10% fetal bovine serum (FBS) (Avantor Seradigm, #97068–085), non-essential amino acids (Thermo Fisher Scientific, #11140–050), and 1x Penicillin-Streptomycin (Corning Inc., #30-002-CI) antibiotic. All cells were incubated at 37°C and in the presence of 5% CO_2_.

### Sequence analysis and site-directed mutagenesis

Clustal Omega was used to generate multiple sequence alignments of flavivirus C protein and M protein regions [70]. Subsequently, Jalview software (University of Dundee, Scotland) was used to analyze and annotate the alignments [71]. Surface exposed residues of C α3 from the ZIKV C crystal structure (PDB: 5YGH) were selected for substitution to alanine using site-directed mutagenesis using Phusion DNA polymerase (New England Biolabs, #MO5305) and primers shown in S1 Table (IDT DNA Inc.). The reactions were treated with DpnI (New England Biolabs, R0176) followed by transformation of NEB Stable (New England Biolabs) *E*. *coli* cells and selection in Lysogeny Broth (LB) agar plates containing 100 ug/ml Ampicillin. Plasmid DNA was purified from isolated colonies using the EconoSpin spin column miniprep kit (Epoch, #2160–250) and sequenced to confirm the mutations at the Huck Genomics Core facility at Pennsylvania State University.

### Transfection, *in vitro* transcription of cDNA clones, and electroporation

Plasmid DNA representing wild-type and mutant ZIKV cDNA were transfected into 2.4×10^5^ HEK-293T cells grown on 24-well culture plates using Transfection Grade Linear Polyethylenimine Hydrochloride (PEI-Max) (Polysciences, #49553-93-7). Media over the cells were replaced with OptiMEM (Thermo Fisher Scientific, #22600–050) 20 min before transfection. The cDNA clone (250ng) was incubated with PEI-Max in Opti-MEM for 20 min and then added to the cells. After 14–16 hours, transfection media were replaced with DMEM supplemented with 10% FBS and incubated at 37°C. Cell culture supernatants were collected 96 hours post transfection (h.p.t.) and stored at -80°C or used to infect Vero E6 cells for fluorescence focus assays and plaque assays. Wild-type and mutant cDNA clones of DENV were digested with *Xba*I and *in vitro* transcribed using T7 RNA polymerase (New England Biolabs, #MO251S) as described previously [72]. Approximately 10 μg of *in vitro* transcribed viral RNA was electroporated into 2.8×10^6^ BHK-15 cells using the BioRad Gene Pulser Xcell system in 0.2 mm cuvettes (BioRad, #1652086). Electroporated cells were incubated at 37°C and in the presence of 5% CO_2_. Supernatants were collected after seven days and stored at -80°C or used to infect BHK-15 cells for plaque assays.

### Microscopy and image acquisition

Fluorescence micrographs ZIKV infected Vero E6 cells were acquired at 10x magnification using DsFi3 camera fitted with Nikon A1R microscope using green excitation:488 nm and emission:500–550 nm filters. All confocal images were acquired using a Nikon A1R confocal microscope with heated 60x oil immersion objective and 1.4 numerical aperture. Live imaging was conducted in a live imaging chamber (Tokai Hit, Fujinomiya, Shizuoka Prefecture, Japan) supplied with 5% CO_2_ maintained at 37°C. Laser and emission band-passes used were as follows: blue, excitation of 405 nm and emission of 425–475 nm; green, excitation of 488 nm and emission of 500–550 nm; red, excitation of 561 nm and emission of 570–620 nm; and far-red, excitation of 640 nm and emission of 660–740 nm. Nikon NIS Elements software was used for all image acquisition and analysis. Nonlinear lookup tables (LUTs) were used to adjust brightness and contrast for clarity.

### Fluorescence analysis and plaque assay

For fluorescence focus analysis, serial 1:10 dilutions of wild-type or mutant ZIKV_venus_ diluted in MEM were used to infect 4.0×10^4^ Vero E6 cells grown on 96-well plates. The relative size of the fluorescent foci was recorded, and representative images were acquired at 5 days post-transfection. All experiments were performed in triplicate. Plaque assays for ZIKV and DENV were performed using Vero E6 and BHK-15 cells, respectively. Cell monolayers grown on 24-well plates were infected with serial dilutions of viruses prepared in a dilution media of MEM supplemented with 10 mM HEPES (N-2-hydroxyethylpiperazine-N’-2-ethanesulfonic acid; Sigma Aldrich, #HO887). Plates were incubated at room temperature on a rocker for 10 minutes, then transferred to a 37°C incubator for 50 minutes. After incubation, cells were overlayed with DMEM (ZIKV) or MEM (DENV) containing 1.5% colloidal cellulose (Sigma-Aldrich, #435244) and 1% FBS, and incubated at 37°C. After a 6-day (ZIKV) or 7-day (DENV) incubation period, cells were fixed with neutral buffered formalin (10% formaldehyde containing 0.004 g/ml NaH_2_PO_4,_ 0.0065 g/ml Na_2_HPO_4_, pH 7.4) for one hour. Fixing solution and overlay were aspirated and cells were washed with Phosphate buffered Saline (PBS) pH 7.4. Subsequently, the cells were stained with 0.05% Crystal violet stain (Sigma-Aldrich, #V5265) in 20% ethanol for 20 minutes. After washing with water, virus plaques were counted, and titers were calculated. All experiments were performed in triplicate.

### Bacterial expression and purification of ZIKV C proteins

Using ligation-independent cloning, open reading frames coding for wild-type or mutant ZIKV C proteins were introduced into bacterial expression plasmid (Addgene, #29708) to express the C protein as N-terminal Maltose binding protein (MBP) fusions (MBP-C). The clones were confirmed by Sanger sequencing, and the plasmids were subsequently transformed into *E*. *coli* BL-21 bacterial cells. Cultures of BL-21 cells transformed with the expression plasmid were grown in 2xYT broth containing 30 μg/ml kanamycin to OD_600_ of 1 at 32°C, and protein expression was induced by adding 0.2 mM IPTG. The cultures were then shaken overnight at room temperature, and cells were pelleted by centrifugation at 15,000 ×g for 15 minutes in a JLA 16.250 rotor in a Beckman Coulter Avanti JE centrifuge. For protein purification, cell pellets were resuspended in 1x PBS and sonicated using a Branson Sonifier-250. The lysates were clarified by centrifugation at 15,000 ×g for 20 minutes using JA 14.50 rotor in the Beckman Coulter Avanti JE centrifuge. Supernatants containing MBP-C fusion proteins were incubated with amylose resin (New England Biolabs, #E8021S) for 60 min at 4°C. Protein-bound resin was loaded on to a gravity column and washed with 20 column volumes of 1x PBS containing 1 M NaCl, followed by 20 column volumes of 1x PBS to remove impurities. The MBP-C fusion proteins were eluted in 2 ml fractions with PBS containing 25 mM D maltose (Fisher Scientific, #6363-53-7). Aliquots of elution fractions were mixed with SDS-loading dye, incubated at 95°C for 5 minutes to denature the protein, and evaluated by sodium dodecyl sulfate–polyacrylamide gel electrophoresis (SDS-PAGE) using self-prepared 11.5% acrylamide-bisacrylamide (Fisher Scientific, #BP 1408–1) gels. Fractions containing a 54 kDa band corresponding to the MBP-C fusion protein were pooled, diluted with water to achieve a final salt concentration of 50 mM, and loaded onto a HiTrap-Heparin-HP affinity column (Cytiva 17-0407-01) connected to AKTA pure 25M fast protein liquid chromatography system operating with Unicorn 7.1 software (GE Healthcare). After washing the column with 3 column volumes of PBS with 60 mM NaCl, a 60 mM to 1 M NaCl salt gradient was applied. MBP-C fusion proteins were eluted at an ion concentration of approximately 800 mM NaCl (~75 mS/cm). The molecular weight of both MBP-C fusion proteins was around 54 kDa as determined by SDS-PAGE.

### Electrophoretic mobility shift assay (EMSA)

Purified wild-type or mutant MBP-C protein was buffer exchanged with PBS using Millipore 30 kDa concentrator to a final protein concentration of 0.2 mg/ml. A gene fragment representing the 5’UTR and C of ZIKV cloned under the T7 promoter was used to generate RNA via *in vitro* transcription using T7 RNA polymerase (New England Biolabs, #MO2515), and the RNA was purified using an RNA clean and concentrator kit (Zymo Research, #R1015). Binding reactions were prepared in which purified RNA and protein were mixed at increasing molar ratios in a buffer containing 20 mM Tris pH 7.4, 50 mM NaCl, and 10 mM MgCl_2_. The binding reactions were allowed to occur at room temperature for 20 min and then separated on a 6% Acrylamide gel prepared in TBE-glycerol buffer (89 mM Tris base, 89 mM boric acid, 2 mM EDTA, and 8% glycerol). The gel was stained in ethidium bromide for 10 min, then washed in deionized water, imaged using the BioRad ChemiDoc XRS+ Molecular imager, and analyzed with Image lab software. All buffers were prepared with Diethyl pyrocarbonate (DEPC, Sigma # D5758) treated water to prevent RNA degradation.

### Glutaraldehyde crosslinking assay

Purified wild-type and mutant MBP-C proteins at a concentration of 0.16 mg/ml in PBS were mixed with increasing concentrations of glutaraldehyde (Sigma Aldrich) in 50 μL reaction volumes. The reaction mix was incubated at room temperature for 20 min, and reactions were stopped by adding Tris pH 8 to 50 mM final concentration. The samples were mixed with SDS loading dye, heated to 70°C for 5 min, and separated on a 10% acrylamide gel. The gel was stained with Coomassie blue, and the image was acquired using the BioRad ChemiDoc XRS+ Molecular imager.

### Subcellular localization of ZIKV C protein

The sequence corresponding to the wild type, or mutant ZIKV C protein was cloned into a CMV promoter-driven pcDNA 3.1(+) mammalian expression vector with an N-terminal mCherry tagged fusion protein. Plasmid DNAs were transfected into JEG-3 cells grown on a 35 mm glass-bottomed dish (IBIDI, #81218–200). Cells were co-transfected with a plasmid coding for GFP-tagged nucleolin (Addgene, #28176) to determine the colocalization of C protein with the nucleoli. The transfected cells were stained with a lipid droplet stain monodansylpentane (MDH) (Abgent, #SM1000A) to analyze the colocalization of C protein to lipid droplets. The cells were incubated with the stain for 30 min at 37°C, and the excess stain was removed by washing thrice with 1x PBS. Media were replaced with Opti-MEM, and confocal images were acquired.

### Immunofluorescence (IF) analysis

JEG-3 cells were grown on 0.13–0.16 mm thick glass coverslips (Thermo Fisher Scientific, #12-545-80) on 24-well plates. When the cell confluency reached 50–80%, cells were transfected with wild-type or mutant ZIKV plasmid cDNA clones using lipofectamine 2000 (Invitrogen, #11668–019). At six hours post-transfection, media were replaced with DMEM/F-12 supplemented with 2% FBS and incubated for 48 h at 37°C and 5% CO_2_. Cells were washed with PBS and fixed with 3.7% paraformaldehyde in PBS for 15 min at room temperature. Next, the cells were washed thrice with PBS and subsequently permeabilized with 0.05% Triton X-100 (VWR, #0694) for 15 min at room temperature. Cells were washed thrice with PBS to remove the detergent and then blocked with PBS containing 10 mg/ml bovine serum albumin (BSA) (Sigma, #A7906) for 1 h at room temperature. Cells were then probed with either combination of primary antibodies against ZIKV C protein (1:150 dilution of rabbit polyclonal ZIKV C, GeneTex, #GTX134186), and Golgi (1:100 dilution of mouse polyclonal anti-Giantin (Abcam, #ab37266), or ZIKV C and flavivirus dsRNA (1:200 dilution of mouse monoclonal anti-dsRNA, Thermo Fisher Scientific, #10010500) for overnight at 4°C. Primary antibodies were removed, cells were washed thrice with PBS and probed with secondary antibodies Tetramethylrhodamine isothiocyanate (TRITC) goat anti-mouse (Thermo Fisher Scientific) diluted 1:200 in BSA and AlexaFluor-647 far-red goat anti-rabbit secondary antibody (Thermo Fisher Scientific, #A32728) diluted 1:100 in BSA. After 1 h incubation at room temperature, secondary antibodies were removed, and cell nuclei were stained with Hoechst stain (Invitrogen) diluted to 0.2 μg/ml in PBS for 15 min. Cells were then washed one final time in PBS before mounting on glass slides with 1.2 mm thickness (VWR, 48300–026) using FluorSave (Millipore, #345789), and confocal images were acquired.

### Quantitative reverse transcription PCR (qRT-PCR)

Virus particles from culture supernatants were purified as described for western blot analysis. The virus pellets were resuspended in 200 μl of PBS, and RNA was isolated using the GenCatch viral RNA miniprep purification kit (Epoch Life Science, 18–60050). All qRT-PCR experiments were performed using purified viral RNA and primers aligning to ZIKV M protein and ZIKV E protein (S1 Table) on the ZIKV genome as previously described [73], using Power SYBR-Green RNA to C_T_ 1-step kit from Applied Biosciences (#4389986). The number of RNA molecules was estimated from C_T_ values calculated using a standard curve generated from known concentrations of *in vitro* transcribed ZIKV RNA molecules. All reactions were carried out in triplicate using the Applied Biosystems QuantStudio 3 PCR system.

### Western blot analysis

Viruses from culture supernatants were purified via ultracentrifugation, and western blot analyses were performed to detect virus particles released from infected cells. Briefly, Vero E6 cells were grown on T75 flasks and infected with wild-type virus, mutant virus, or mock-infected for 60 min at 37°C in the presence of 5% CO_2._ Cells were washed twice with PBS, and media were replaced. Cell culture supernatants were collected 2 d.p.i. by centrifugation at 15,000 ×g for fifteen min. This supernatant was loaded on a 1 ml cushion of 25% Sucrose in Tris-NaCl-EDTA (TNE) buffer in 13.2 ml round bottom tubes (Beckman Coulter, #344059). The samples were spun at 100,000 ×g for two hours in SW 41 Ti rotor using a Beckman Coulter TLX-Optima ultracentrifuge to pellet viral particles. The virus pellet was resuspended in 150 μl of 1x SDS loading dye, heated to 90°C for 5 min, and separated in a 12.5% acrylamide (Fisher Scientific, #BP1408) gel (self-prepared) for electrophoresis. Proteins were transferred from the gel to a PVDF membrane (Cytiva Amersham, #10600021) using a BioRad Trans-Blot Turbo transfer system. The membrane was washed thrice in 1x Tris-buffered saline with 0.1% Tween 20 (VWR, #0777) (TBST) and blocked overnight at 4°C in LI-COR Intercept Blocking buffer (LICOR #927–60001). The blot was probed with primary antibody against ZIKV C (GeneTex, #GTX134186) at a 1:1000 dilution in blocking buffer and incubated overnight at 4°C with rocking. Subsequently, the membrane was washed four times with TBST and probed with goat anti-rabbit secondary antibody conjugated with horse radish peroxidase (Sigma Aldrich, A0545) at a 1:1000 dilution in the blocking buffer. After 1 h incubation at RT with rocking, the blot was developed using Super Signal WestPico PLUS Chemiluminescent substrate (Thermo Fisher Scientific, #34577) and imaged using the BioRad ChemiDoc XRS+ Molecular imager and analyzed with Image lab software.

### Revertant screening

Supernatants from HEK 293-T cells transfected with ZIKV_venus_ containing the C_N67A_ mutation were collected (passage 0) and used to infect Vero E6 cells grown on 6-well plates and incubated for 6 days. The supernatant was then harvested to infect new Vero E6 cells (passage 1). This process was repeated until the >100 cell fluorescent clusters were observed (passage 5). At this point, cells were harvested, and total RNA was extracted using a Zymo Research Quick-RNA miniprep RNA purification kit. From extracted RNA, regions corresponding to the C and prM (nt 1–1030), E (nt 693–1816 and nt 1212–2511), NS2A (nt 3545–4399), and NS3 (nt 4377–5272 and nt 5242–6528) were reverse transcribed using OneTaq One step 2x master mix (New England Biolabs, #M04825) using primers as listed in S1 Table. Amplicons were solution purified (Epoch Life Science, #2360050) and sequenced at the Huck Genomic Core facility at Pennsylvania State University. Sequences were analyzed using Serial Cloner and FinchTV (Geospiza Inc.) to identify second site mutations.

### Virus growth kinetics

For growth kinetic analyses of ZIKV, Vero E6 cells grown to 80% confluence on 6-well plates were infected with wild-type or mutant ZIKV at a multiplicity of infection (MOI) of 1.0 for one hour at 37°C. Cells were washed with PBS, and media was replaced with DMEM supplemented with 10% FBS, and incubation was continued at 37°C with 5% CO_2_. After 24, 48, 72, and 96 hours, 200 μl cell culture supernatant samples were collected and replenished with 200 μl fresh growth medium. Viruses released into the media were quantified using plaque assays on Vero E6 cells as described above. For DENV growth kinetics analysis, BHK-15 cells at 95% confluence grown on 24-well plates were infected with wild-type or mutant DENV at an MOI of 0.01 for one hour at 37°C. Cells were washed with PBS, and media were replaced with MEM supplemented with 10% FBS. After 24, 48, 72, and 96 hours, 50 μl supernatant samples were collected from the infected cells and replaced with fresh growth medium. Virus particles released into the media were quantified using plaque assays on BHK-15 cells as described elsewhere.

## Supporting information

S1 Fig Predicted structures of alanine substitutions. AlphaFold predictions of (A) wild type ZIKV C, (B) C_I66A_, (C) C_N67A_, (D) C_R68A_, (E) C_S71A_, colored by confidence level (color key top right). All figures are overlayed with the crystal structure of wild type ZIKV C (PDB: 5YGH) (pale green).
(TIF)

S2 Fig Size Exclusion Chromatography. A_280_ elution profiles of (A) MBP-C_N67A_, (B) MBP-C, and (C) MBP ran at 0.5 ml/min on a Superdex 200 GL column (Cytiva) equilibrated with PBS containing 0.5M NaCl.
(TIF)

S3 Fig Comparison of flavivirus capsid structures. Overlayed structures of ZIKV C (PDB: 5YGH) (pale green) with (A) DENV C NMR structure (PDB: 1R6R), (B) WNV C crystal structure (PDB: 1SFK) (orange), and (C) JEV C crystal structure (PDB: 5OW2) (pink). Zoomed box in each image shows α3 residue orientations as compared to ZIKV C.
(TIF)

S1 Table List of primers used for mutagenesis and cloning. Mutated codons are underlined.
(DOCX)
