# Supplementary figures and images for: Identification of a critical role for ZIKV capsid α3 in virus assembly and its genetic interaction with M protein
Source: PLoS Negl Trop Dis. 2024 Jan 2;18(1):e0011873. doi: 10.1371/journal.pntd.0011873 (PMC10786401; doi:10.1371/journal.pntd.0011873)

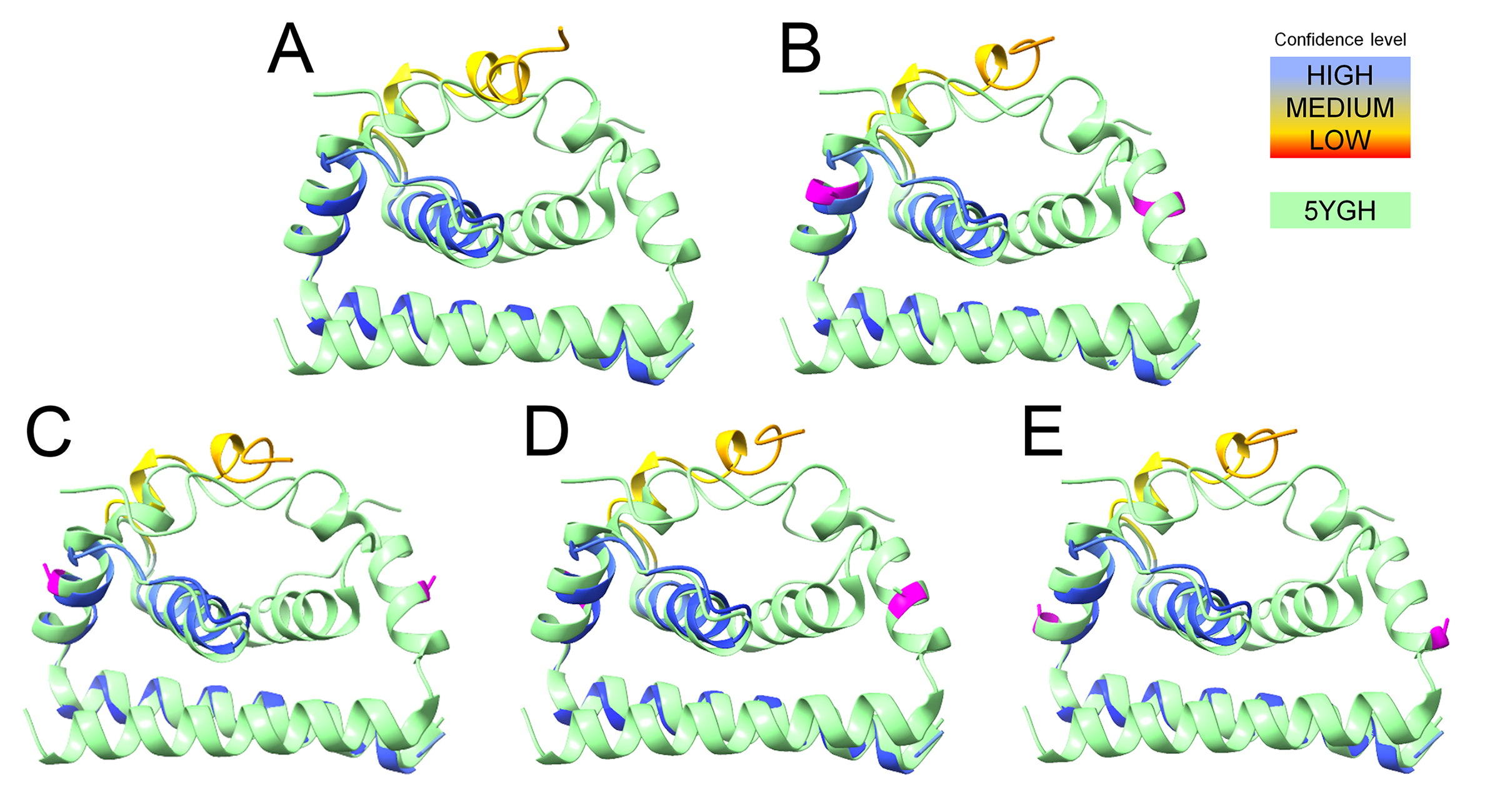

Supplement: S1 Fig — AlphaFold predictions of (A) wild type ZIKV C, (B) CI66A, (C) CN67A, (D) CR68A, (E) CS71A, colored by confidence level (color key top right). All figures are overlayed with the crystal structure of wild type ZIKV C (PDB: 5YGH) (pale green). (TIF) [file pntd.0011873.s001.tif]

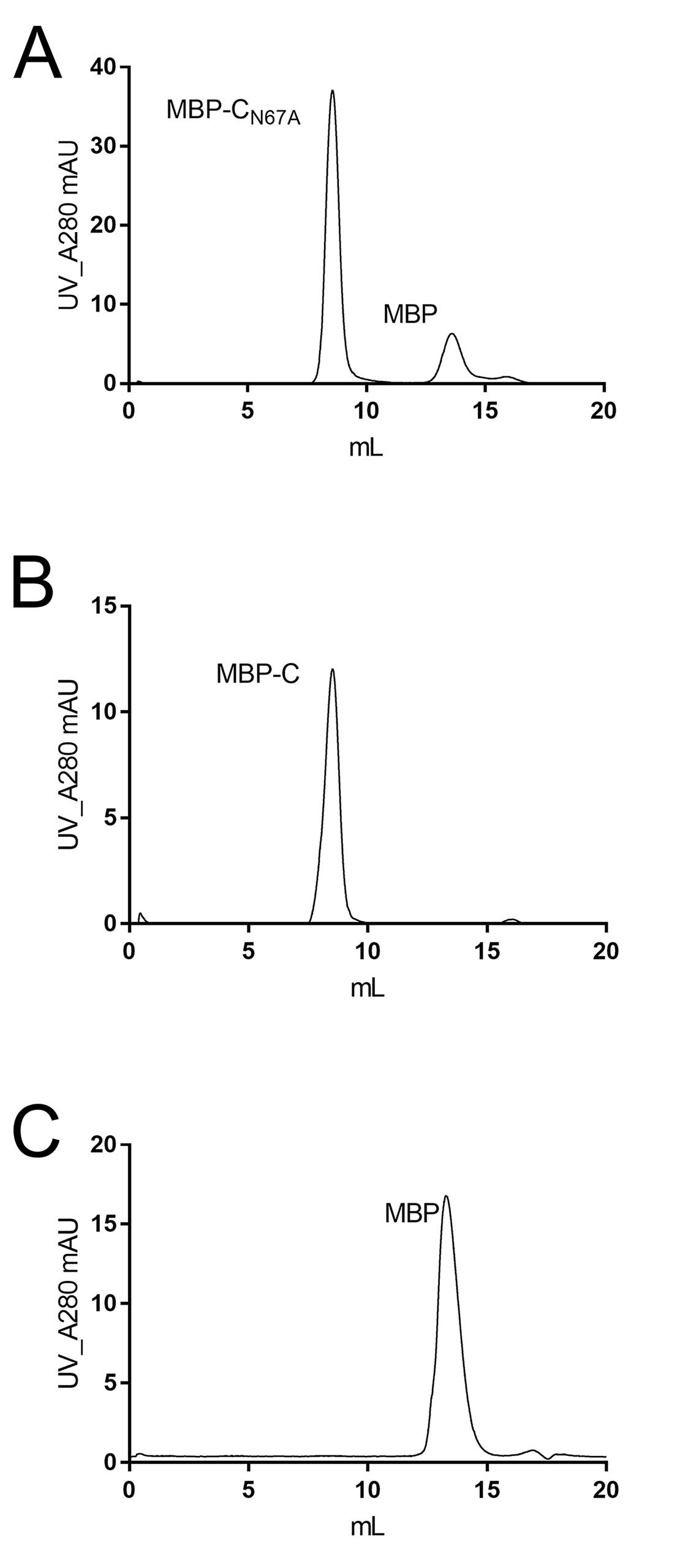

Supplement: S2 Fig — A280 elution profiles of (A) MBP-CN67A, (B) MBP-C, and (C) MBP ran at 0.5 ml/min on a Superdex 200 GL column (Cytiva) equilibrated with PBS containing 0.5M NaCl. (TIF) [file pntd.0011873.s002.tif]

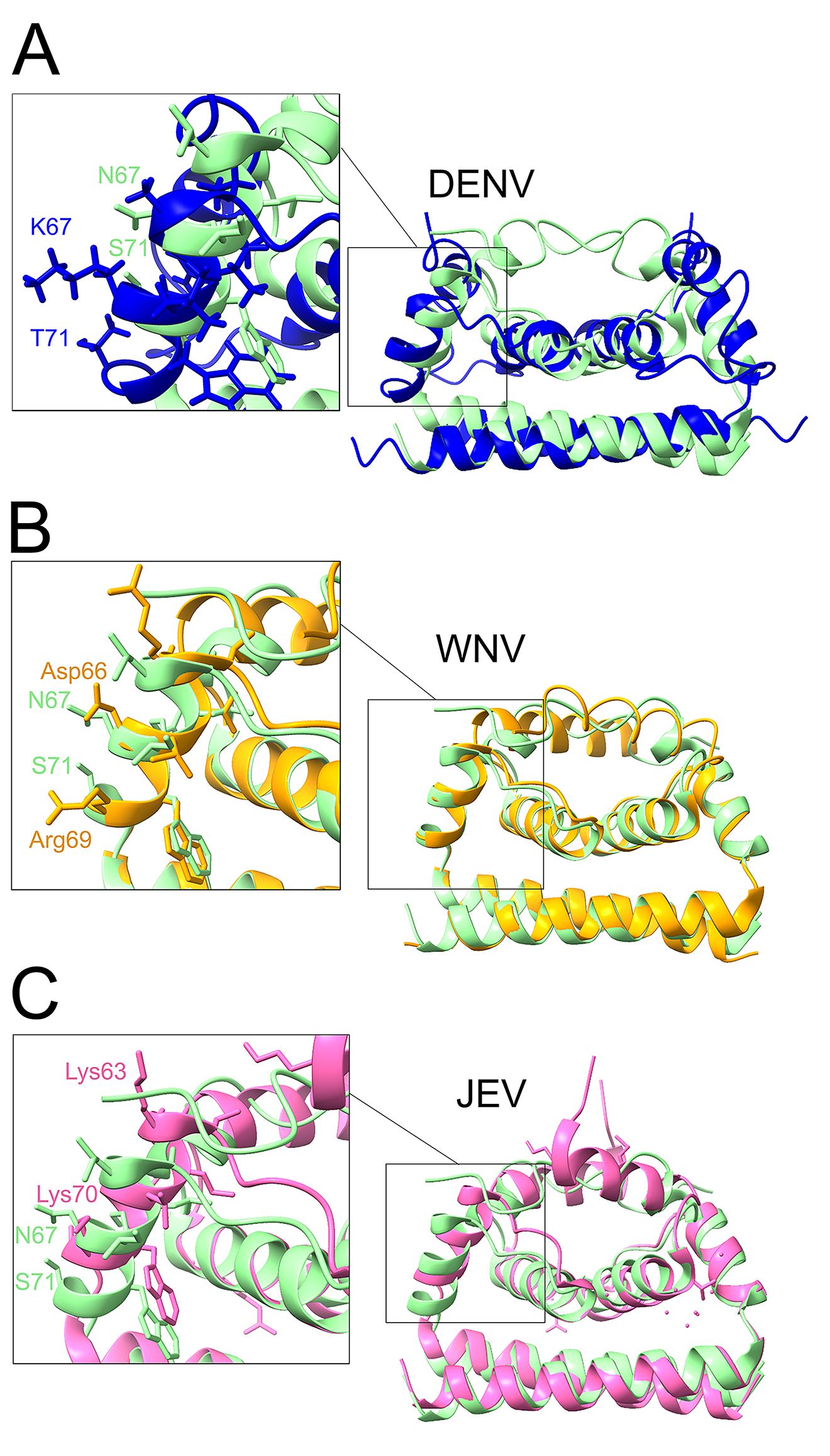

Supplement: S3 Fig — Overlayed structures of ZIKV C (PDB: 5YGH) (pale green) with (A) DENV C NMR structure (PDB: 1R6R), (B) WNV C crystal structure (PDB: 1SFK) (orange), and (C) JEV C crystal structure (PDB: 5OW2) (pink). Zoomed box in each image shows α3 residue orientations as compared to ZIKV C. (TIF) [file pntd.0011873.s003.tif]
