# Supplementary material for: Identification of a critical role for ZIKV capsid α3 in virus assembly and its genetic interaction with M protein
Source: PLoS Negl Trop Dis. 2024 Jan 2;18(1):e0011873. doi: 10.1371/journal.pntd.0011873 (PMC10786401; doi:10.1371/journal.pntd.0011873)
Supplement: S1 Table — Mutated codons are underlined. (DOCX) [file pntd.0011873.s004.docx]

**SUPPLEMENTARY INFORMATION**

**Table S1.** **List of primers used for mutagenesis and cloning.**

| **Virus, protein** | **Purpose** | **Forward (F) and reverse (R) Primer sequences** |
| --- | --- | --- |
| ZIKV, C | I66A mutation | (F) TGGGCCTTGCTAACAGATGGGGTTCCGTGGGGAAAAAAG  (R) ATCTGTTAGCAAGGCCCAGTGATGGCTTGATTGCTG |
| ZIKV, C | N67A mutation | (F) TGGGCCTTATCGCTAGATGGGGTTCCGTGGGGAAAAAAG  (R) ATCTAGCGATAAGGCCCAGTGATGGCTTGATTG |
| ZIKV, C | R68A mutation | (F) TTATCAACGCGTGGGGTTCCGTGGGGAAAAAAGAG  (R) AACCCCACGCGTTGATAAGGCCCAGTGATGGCTTG |
| ZIKV, C | S71A mutation | (F) ATGGGGTGCTGTGGGGAAAAAAGAGGCTATGGAAATAA  TAAAG  (R) TCCCCACAGCACCCCATCTGTTGATAAGGCCCAGTGAT |
| ZIKV, C | Combination mutant (Cm)  [_66_INRWGS_71_/  _66_AAAWGA_71_] | (F) TGCTGCAGCCTGGGGAGCTGTGGGGAAAAAAGAGGCTATG  (R)AGCTCCCCAGGCTGCAGCAAGGCCCAGTGATGGCTTGATTGC |
| ZIKV, C | N67M mutation | (F) TGGGCCTTATCATGAGATGGGGTTCCGTGGGGAAAAAAG  (R) ACCCCATCTCATGATAAGGCCCAGTGATGGCTTGATTG |
| ZIKV, C | N67L mutation | (F) TGGGCCTTATCCTGAGATGGGGTTCCGTGGGGAAAAAAG  (R) ATCTCAGGATAAGGCCCAGTGATGGCTTGATTG |
| ZIKV, C | N67G mutation | (F) TGGGCCTTATCGGTAGATGGGGTTCCGTGGGGAAAAAAG  (R) ATCTACCGATAAGGCCCAGTGATGGCTTGATTG |
| ZIKV, C | N67R mutation | (F) TGGGCCTTATCAGGAGATGGGGTTCCGTGGGGAAAAAAG  (R) ATCTCCTGATAAGGCCCAGTGATGGCTTGATTG |
| ZIKV, C | N67K mutation | (F) TGGGCCTTATCAAGAGATGGGGTTCCGTGGGG  (R) ATCTCTTGATAAGGCCCAGTGATGGCTTGATTG |
| ZIKV, M | F37A mutation | (F) ACTGGATAGCCAGGAACCCCGGGTTTGCGCTAG  (R) GTTCCTGGCTATCCAGTTTTCAACCTTGATCAA |
| ZIKV, M | F37I mutation | (F) ACTGGATAATCAGGAACCCCGGGTTTGCGCTAG  (R) GTTCCTGATTATCCAGTTTTCAACCTTGATCAA |
| ZIKV, M | F37W mutation | (F) ACTGGATATGGAGGAACCCCGGGTTTGCGCTAG  (R) GTTCCTCCATATCCAGTTTTCAACCTTGATCAA |
| ZIKV, M | F37L mutation | (F) ACTGGATACTCAGGAACCCCGGGTTTGCGCTAG  (R) GTTCCTGAGTATCCAGTTTTCAACCTTGATCAA |
| DENV, C | T62A mutation | (F) TCCCACCAGCAGCAGGGATATTGAAGAGATG  (R) TCCCTGCTGCTGGTGGGATTGTTAGGAAACG |
| DENV, C | I65A mutation | (F) AGCAGGGGCATTGAAGAGATGGGGAAC  (R) TCTCTTCAATGCCCCTGCTGTTGGTGGGATTG |
| DENV, C | L66A mutation | (F) AGGGATAGCAAAGAGATGGGGAAC  (R) ATCTCTTTGCTATCCCTGCTGTTGGTGGGATTG |
| DENV, C | K67A mutation | (F) ATATTGGCAAGATGGGGAACAATTAAAAAATCAAAAGC  (R) ATCTTGCCAATATCCCTGCTGTTGGTGG |
| DENV, C | T71A mutation | (F) ATGGGGTGCTGTGGGGAAAAAAGAGGCTATGGAAATAATAAAG  (R) TCCCCACAGCACCCCATCTGTTGATAAGGCCCAGTGATG |
| DENV, M | L37A mutation | (F) ACTTGGATCGCAAGACATCCAGGCTTCACCATGATGG  (R) ATGTCTTGCGATCCAAGTTTCAATTCTCTGGACATG |
| DENV, M | L37F mutation | (F) ACTTGGATCTTCAGACATCCAGGCTTCACCATGATGG  (R) ATGTCTGAAGATCCAAGTTTCAATTCTCTGGACATG |
| DENV, C | Combination mutant (Cm) [_62_TAGILKRWGT_71_/  _62_AAGAAARWGA_71_] | (F) AGGGGCAGCCGCAAGATGGGGAGCGATTAAAAAATCAAAAGC  (R) TCCCCATCTTGCGGCTGCCCCTGCCGCTGGTGGGATTGTTAG |
| DENV, C | Double mutant (Dm-1)  [_66_LK_67_/_66_AA_67_] | (F) AGGGATAGCAGCTAGATGGGGAACAATTAAAAAATC  (R) ATCTAGCTGCTATCCCTGCTGTTGGTGGGATTGTTAG |
| DENV, C | Double mutant (Dm-2) [_67_KRWGT_71_/  _67_ARWGA_71_] | (F) TTGGCAAGATGGGGAGCTATTAAAAAATCAAAAGC  (R) AGCTCCCCATCTTGCCAATATCCCTGCTGTT |
| DENV, C | ZIKV a3 helix swap (Sw) [_62_TAGILKRWGT_71_/  _62_SLGLINRWGSVI_71_] | (F)TGGGCCTTATCAACAGATGGGGTTCCGTGATTAAAAAATCAAAAGCT  (R)ACCCCATCTGTTGATAAGGCCCAGTGATGGTGGGATTGTTAGGAAACG |
